# Improvement of structural efficiency in metals by the control of topological arrangements in ultrafine and coarse grains

**DOI:** 10.1038/s41598-021-96930-3

**Published:** 2021-08-31

**Authors:** Abdallah Shokry, Aylin Ahadi, Per Ståhle, Dmytro Orlov

**Affiliations:** 1 Department of Mechanical Engineering, Faculty of Engineering, Fayoum University, Fayoum, 63514 Egypt; 2 Division of Solid Mechanics, LTH, Lund University, Box 118, 22100 Lund, Sweden; 3 Smart Engineering Systems Research Center (SESC), Nile University, Shaikh Zayed City, Giza, 12588 Egypt; 4 Division of Mechanics, LTH, Lund University, Box 118, 22100 Lund, Sweden; 5 Division of Materials Engineering, LTH, Lund University, Box 118, 22100 Lund, Sweden

**Keywords:** Mechanical properties, Metals and alloys

## Abstract

Improvement of structural efficiency in various materials is critically important for sustainable society development and the efficient use of natural resources. Recently, a lot of attention in science and engineering has been attracted to heterogeneous-structure materials because of high structural efficiency. However, strategies for the efficient design of heterogenous structures are still in their infancy therefore demanding extensive exploration. In this work, two-dimensional finite-element models for pure nickel with bimodal distributions of grain sizes having ‘harmonic’ and ‘random’ spatial topological arrangements of coarse and ultrafine-grain areas are developed. The bimodal random-structure material shows heterogeneities in stress–strain distributions at all scale levels developing immediately upon loading, which leads to developing concentrations of strain and premature global plastic instability. The bimodal harmonic-structure material demonstrates strength and ductility significantly exceeding those in the bimodal random-structure as well as expectations from a rule of mixtures. The strain hardening rates also significantly exceed those in homogeneous materials while being primarily controlled by coarse-grain phase at the early, by ultrafine-grain at the later and by their compatible straining at the intermediate stages of loading. The study emphasises the importance of topological ultrafine-/coarse-grain distributions, and the continuity of the ultrafine-grain skeleton in particular.

## Introduction

Structural properties along with the efficiency of natural and various anthropogenic materials is a challenge virtually on parity with the age of human beings. Its improvement is yet as critically important as never before for the sustainable development of our society and the efficient use of natural resources towards achieving circular economy.

It is well established that structural properties of crystalline materials heavily depend on grain size. From the works of Hall^1^ and Petch^2^ in the early 1950th, a multitude of studies, especially in the area of severe plastic deformation over the past three decades, demonstrated the advantages of grain refinement down to ultra-fine grain (UFG, crystallite or grain size *d* ≤ 1.0 µm) and nano-scale (*d* ≤ 0.1 µm)  ranges. Summaries on major achievements in the area can be found in recent reviews^3–6^. Nevertheless, despite the extreme strength of UFG and nano-metals, their Achilles heel remains to be a very limited ductility compared to coarse-grained (CG, *d* ≥ 10 µm) counterparts^6,7^.

**Figure 1:**
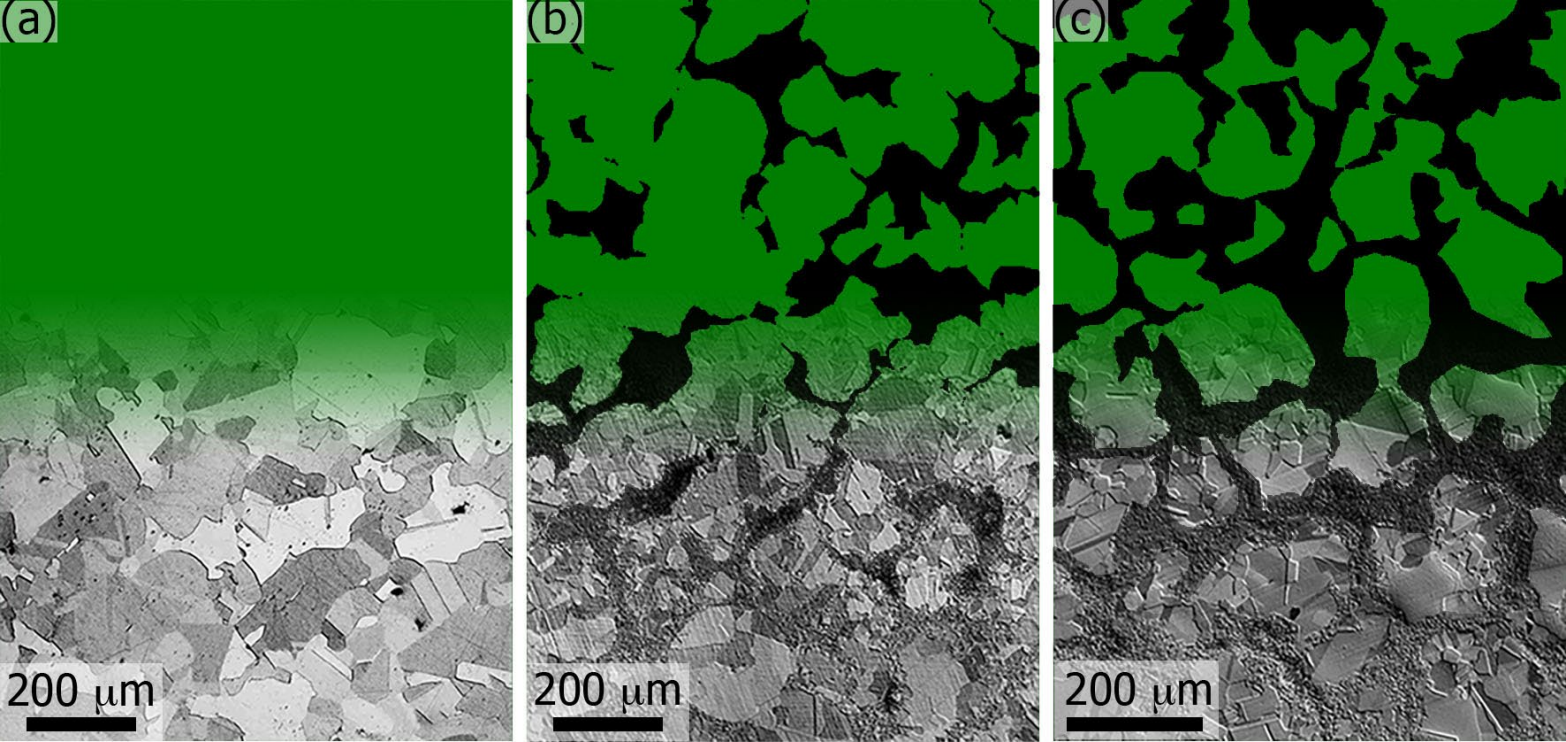
Representative optical micrographs of pure nickel with homogeneous CG (**a**) and heterogeneous bimodal random bR (**b**) and harmonic bHS (c) grain structures; the latter two (**b**,**c**) having a nominal UFG fraction of 40%. Actual (measured) fractions of UFG areas in (**b**,**c**) are *f*_UFG_ = 34% and *f*_UFG_ = 39%, respectively; mean diameters of coarse grains are *d*_CG_ = 26.1 μm in (**a**), *d*_CG_ = 20.4 μm in (**b**), and *d*_CG_ = 21.0 μm in (**c**). The images have gradient transitions from real micrographs at the bottom to segmented areas at the top for CG (green) and UFG (dark-grey) core and shell, respectively. Note disconnected clusters of UFG grains in (**b**) versus virtually continuous UFG skeleton in (**c**).

The most recent strategy in optimising structural efficiency of metals for high strength and high ductility from a grain size perspective is closer to mimicking natural including bio-materials that have heterogeneous structures architected at micro-/nano-scales^8,9^. In particular, the fabrication of heterogeneous structures having gradient and bimodal grain size distributions was proposed recently^10–12^. The efficiency of the latter in improving structural performance was also confirmed in recent analytical studies, for instance in Cu^13^ and Ni^14–16^ as well as in NiTi alloy^17^ and Cu-Nb composite^18^. The latest developments in this direction include fabrication of micro-architected materials with ‘gradient’, ‘heterogeneous-lamella’ and ‘harmonic’ structures^11,19–21^. The latter category is particularly interesting in this context since it offers additional control of three-dimensional (3D) topological arrangement of coarse- and fine-grain areas forming the microstructure. Such materials can be fabricated through a powder metallurgy route^21–23^. Representative real-life microstructures of a model material studied experimentally in^24^ are shown in Fig. 1. This allows ensuring truly high-performance of the bimodal structured materials^11,19,20,25^. Furthermore, harmonic-structure (HS) reduces the variation of properties therefore improving the reproducibility of results^26^.

HS materials have a bimodal grain size distribution and fully controllable 3D topology, which is heterogeneous on micro- but homogeneous on macro-scales. The HS design concept is particularly useful when alloying and other means of microstructure control are restricted by environmental and other requirements. It can also be extended to a variety of materials. However, the detailed understanding of structural behaviour under mechanical loading including phenomenological mechanisms of plastic deformation in HS materials is still missing, as has been emphasised in a recent review^21^. Such knowledge is necessary for elaborating strategies for the efficient design of HS and other heterogenous-structure materials, which demands the development of various analytical and computational models.

Analytical models based on continuum mechanics approaches to investigate the development of strain gradients and back-stress build-up from dislocation pileups (or grain-size)^27^ and precipitate-induced^28^ lattice defects have emerged recently. At the same time, numerical simulation using a finite element (FE) method is useful for studying and visualising the plastic deformation of metals and alloys under mechanical loading^29–32^, including severe plastic deformation processes for the fabrication of bulk UFG materials^4,33–35^. Some efforts in modelling the behaviour of HS materials have also been made recently^16,36–38^ but more investigations in this direction are necessary.

In this paper, we report the numerical investigation of elastic–plastic behaviour in heterogenous materials on the example of pure nickel. FE modelling is used to study the mechanical behaviour of bimodal-structured material having two different morphologies (topological distributions) of UFG and CG areas. A bimodal harmonic-structure (bHS) model, in which CG areas are uniformly distributed within a continuous UFG skeleton, is compared with a bimodal random (bR) distribution model of CG and UFG areas. Phenomenological mechanisms of plastic deformation in materials having heterogeneous grain structure are clarified, while reasons behind the importance of topological UFG/CG distributions and the continuity of the UFG skeleton in particular are revealed. The bR material develops heterogeneities in stress–strain distributions at all scale levels immediately upon loading, which leads to strain concentrations and premature global plastic instability. In consistence with other reports, the bHS material demonstrates strength and ductility significantly exceeding those in the bR as well as expectations from a rule of mixtures, while its strain hardening rates significantly exceed those in homogeneous materials. We show that strain hardening rates in the bHS material are primarily controlled by CG phase at the early, by UFG at the later and by their compatible straining at the intermediate stages of loading.

## Results

In this section, the mechanical behaviour of the bHS and the bR samples is studied at different strains. The different strain levels are selected to study the elastic–plastic transition, partitioning of plastic strain between CG and UFG phases, the accumulation of strain and subsequent strain localisation just after reaching the ultimate strength, resulting in the loss of stability and numerical convergence.

In addition to the aforementioned models of heterogeneous materials, two simulations are made for homogeneous 100% CG and 100% UFG samples using the same geometry and boundary conditions. These are used as benchmarks for comparative analysis.

### Global stress–strain behaviour

**Figure 2:**
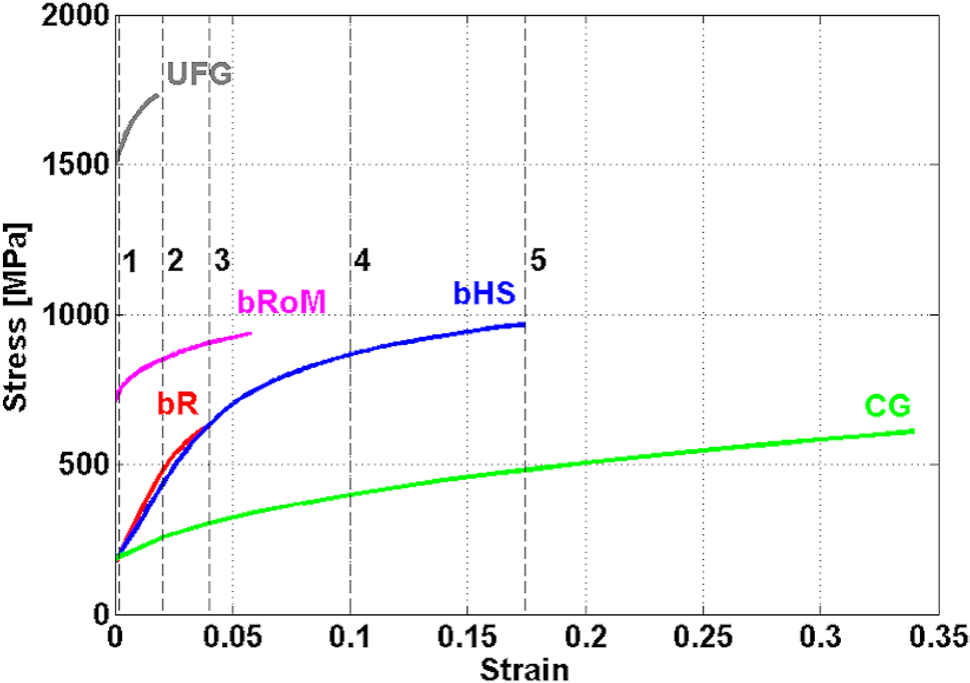
Mean true stress ${\sigma }_{eff}$ versus mean true plastic strain ${\varepsilon }_{eff}$ curves for the simulated bHS and bR models. Calculated curves for bimodal rule of mixtures (bRoM), 100% CG and 100% UFG samples are also included to serve as references. Dashed vertical lines indicate characteristic strain levels.

Figure 2 shows the results of true stress–strain curve calculations for the bHS and bR models along with 100% CG and 100% UFG benchmark materials. The curves for bHS and bR are presented as average values over the full column of unit cells at the section left sides. In addition, a curve for a bimodal Rule of Mixture (bRoM) dependence is included. For the bRoM curve, direct volumetric proportionality equation is used, so that

1 $${\sigma }_{bRoM}\left({\varepsilon }_{eff}\right)={f}_{UFG}{\sigma }_{UFG}\left({\varepsilon }_{eff}\right)+{f}_{CG}{\sigma }_{CG}\left({\varepsilon }_{eff}\right),$$

where ${\sigma }_{bRoM}$ is the standard estimation of true stress in a general mixture of 100% CG and 100% UFG materials that each at the same strain ${\varepsilon }_{eff}$ would carry the true stresses ${\sigma }_{UFG}$ and ${\sigma }_{CG}$. Further, ${f}_{UFG}$ and ${f}_{CG}$ are the volume fractions of the UFG and CG phases, i.e. ${f}_{UFG}=0.4$ and ${f}_{CG}$ = 0.6 in the present study.

The largest presented elongations of the curves mark the final vanishing cross section and loss of convergence. The curve obtained from the rule of mixture bRoM is truncated at the maximum output stress of UFG, at this point the material continues to elongate due to the CG phase.

The simulated curves of homogeneous 100% CG and 100% UFG materials in Fig. 2 reveal very good reproducibility of experimental results, see dotted curves Fig. 10c for comparison. These are expected results confirming the validity of the developed FE model. At the same time, it should be noted that yield strength of both bR and bHS materials is significantly lower than that of bRoM, which is not consistent with experimental results reported in the literature, cf. works of Zhu et al^12^ for broad overview and Orlov and Ameyama^21^ for a more specific review on bHS works. This discrepancy is attributed to the limitations of our FE model at present where rheology of both UFG and CG phases are introduced discretely for elastic and plastic parts of respective stress–strain curves. This apparent weakness of the present model will be resolved in future studies. A potent analytical model along with a numerical approach for elaborating a better rheological model for bHS materials is published recently^16^. While the UFG material has the highest strength and the lowest elongation to failure, the CG one demonstrates the reverse: highest elongations with the lowest strength. These curves can now be used as a benchmark for comparison with other material simulations.

All curves for the heterogeneous materials in the present study reveal intermediate values of strength and ductility characteristics. The bR material demonstrates a minor increase in strength over the CG counterpart along with dramatically reduced uniform elongation, which is still larger than that of the pure UFG material. The bHS model reveals a strain hardening rate very similar to that in bR but 40% higher ultimate strength along with more than three times higher uniform elongation. When compared with 100% CG, the bHS curve still reveals almost two-times lower elongation at almost as high strength.

A very interesting finding can be made from comparing these two curves with one calculated as a ‘rule of mixtures’, Eq. (1), typically used for the upper estimate of strength in composite materials^39^. Compared to that, the bR material is still weaker and less ductile while the bHS demonstrates substantially higher strength along with approximately three times higher elongation. As expected from a weakest link hypothesis, the structural efficiency of a well-ordered HS architecture might be higher than in composites with random distribution of a reinforcing phase.

Based on these observations, five characteristic strain levels are selected for the in-depth analysis of spatial stress and strain distributions in bR and bHS materials. These are indicated in Fig. 2 with five vertical dashed black lines numbered 1 to 5 corresponding to strain levels 0.002 (elastic–plastic transition), 0.02 and 0.04 (beginning of local stress concentration in 100% UFG and bR materials, respectively), 0.1 and 0.174 (developed plastic deformation and beginning of local stress concentration in bHS material, respectively). These are presented and discussed in detail in Supplementary information S1. The section below summarises the most important conclusions from these relevant to the development of local stress and strain gradients. Note the fracture mechanisms are not studied in this work because of the limitations of present models.

### Development of local stress and strain gradients

**Figure 3:**
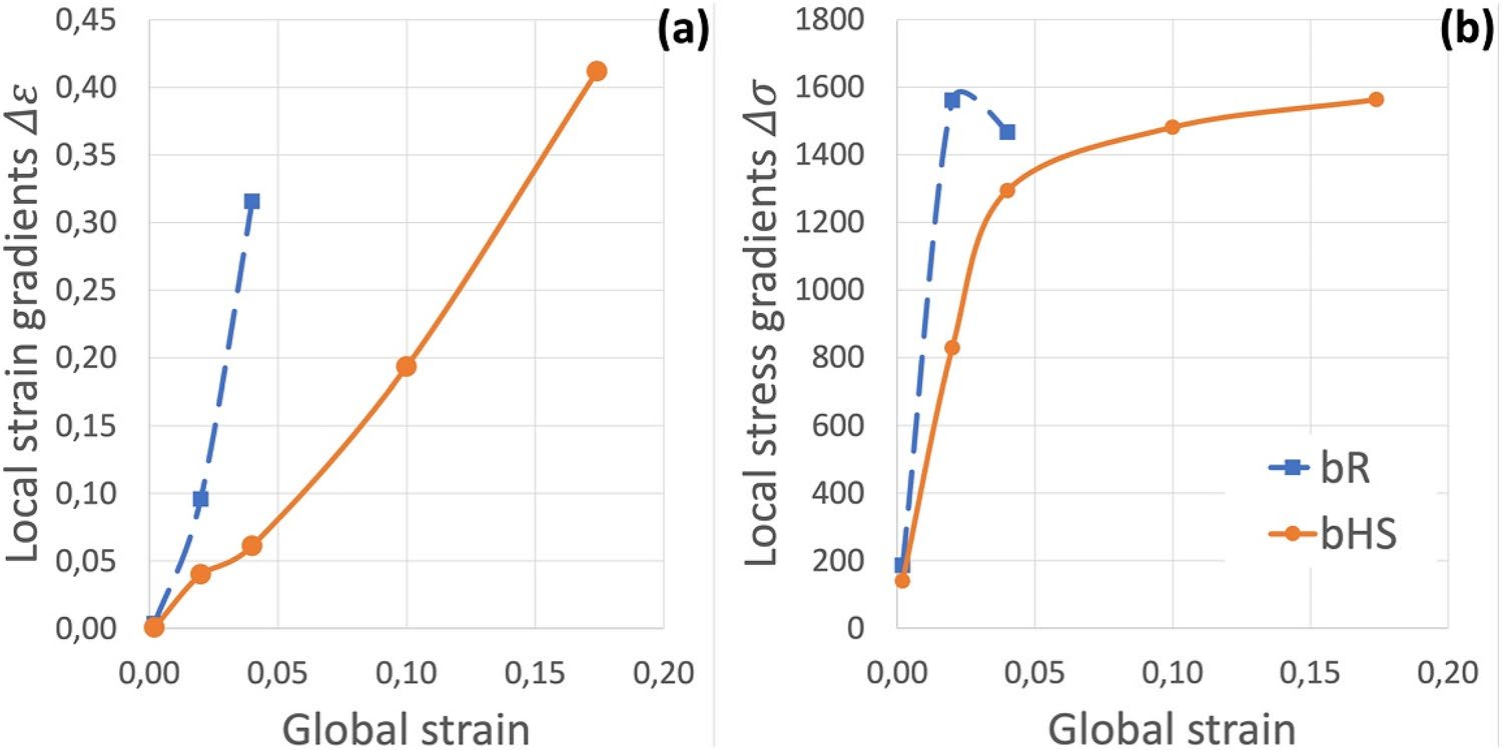
Development of maximum local strain (**a**) and stress (**b**) gradients in bimodal random (bR) and bimodal harmonic-structure (bHS) materials as a function of global strain ${\varepsilon }_{eff}$.

Homogeneous-structure materials, both 100% CG and 100% UFG, deform homogeneously up until necking, i.e. the beginning of global plastic instability. Such a behaviour is typical and therefore will not be discussed in detail here. However, both bimodal heterogeneous-structure materials start developing local gradients in stress and strain distributions from the very beginning of mechanical loading. These are summarised in Fig. 3 for strain (Fig. 3a) and stress (Fig. 3b) distributions, which complements more graphical and comprehensive discussion in the Supplementary information S1.

The material with random grain size distribution bR develops large strain and stress gradients very rapidly, which leads to the global plastic instability (macroscopic sample fracture) already at ${\varepsilon }_{eff}$ = 0.04. At this global strain level in bR material, local strain gradients are at their highest, while the macroscopic fracture leads to an appreciable relaxation of local stress gradients.

By contrast, the material with regular/controlled grain size distribution bHS develops strain and stress gradients gradually. Although development of strain gradients accelerates with the increase of global strain, the development of stress gradients decreases concomitantly. The latter effectively saturates at approximately the same level as in bR material but at a significantly higher level of global strain at ${\varepsilon }_{eff}$ = 0.174. It is also worth noting that the regular distribution of gradients allows the bHS material to tolerate higher strain gradients than the bR.

### Strain tensor components at the onset of macroscopic strain localisation

**Figure 4:**
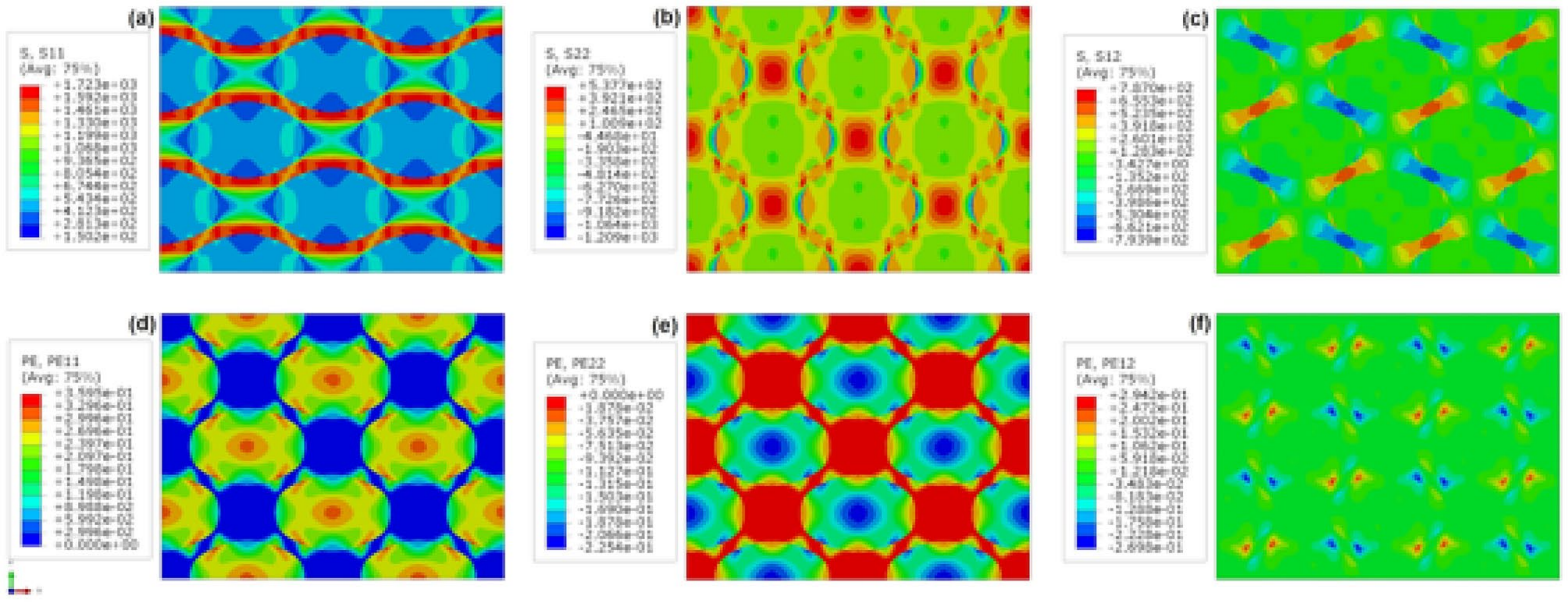
Distribution of normal *x*, *y*, and shear *xy* stresses (**a**–**c**) and plastic strains (**d**–**f**) tensor components at a global strain ${{\varvec{\varepsilon}}}_{{\varvec{e}}{\varvec{f}}{\varvec{f}}}=$ 0.174 in representative cells of bHS material.

Figure 4 shows contour plots for orthogonal components of stress and strain tensors in *x* and *y* directions as well as shear components in *x–y* plane for the indicative four unit cells of bHS sample at global strain ${{\varvec{\varepsilon}}}_{{\varvec{e}}{\varvec{f}}{\varvec{f}}}$ **=** 0.174. The longitudinal stresses in *x* direction coinciding with the direction of specimen tension are tensile only, Fig. 4a. The highest ${\sigma }_{x}$ stresses propagate in horizontal direction exclusively through UFG skeleton as ‘necks’-thick bands. The normal stresses in *y*-direction vary between tensile (positive, predominantly in the UFG skeleton) and compressive (negative, predominantly in the CG areas), Fig. 4b. Sharp gradients in ${\sigma }_{y}$ stress variations can be found at the UFG/CG interfaces propagating within thick bands in vertical direction. The shear stresses are concentrated within the UFG ‘necks’ with peak values in the thinnest region, Fig. 4c. With virtually the same absolute values by modulus, they are positive in 45° counter-clockwise and negative in 45° clockwise direction from applied tension.

The normal strain tensor components are close to ‘0’ in the majority of UFG skeleton, Fig. 4d–f. Longitudinal strain components are all tensile (positive) and highest in the centre of CG areas monotonically decreasing in both the orthogonal directions. At the same time, local maxima of the same magnitude can be found in the vicinity of the UFG ‘necks’. Transverse strains are all compressive (negative) having inverse distribution trend and approximately 37% lower values by modulus compared to the longitudinal stresses. Shear strains are located around the UFG ‘necks’ and have the distribution similar to shear stresses.

**Figure 5:**
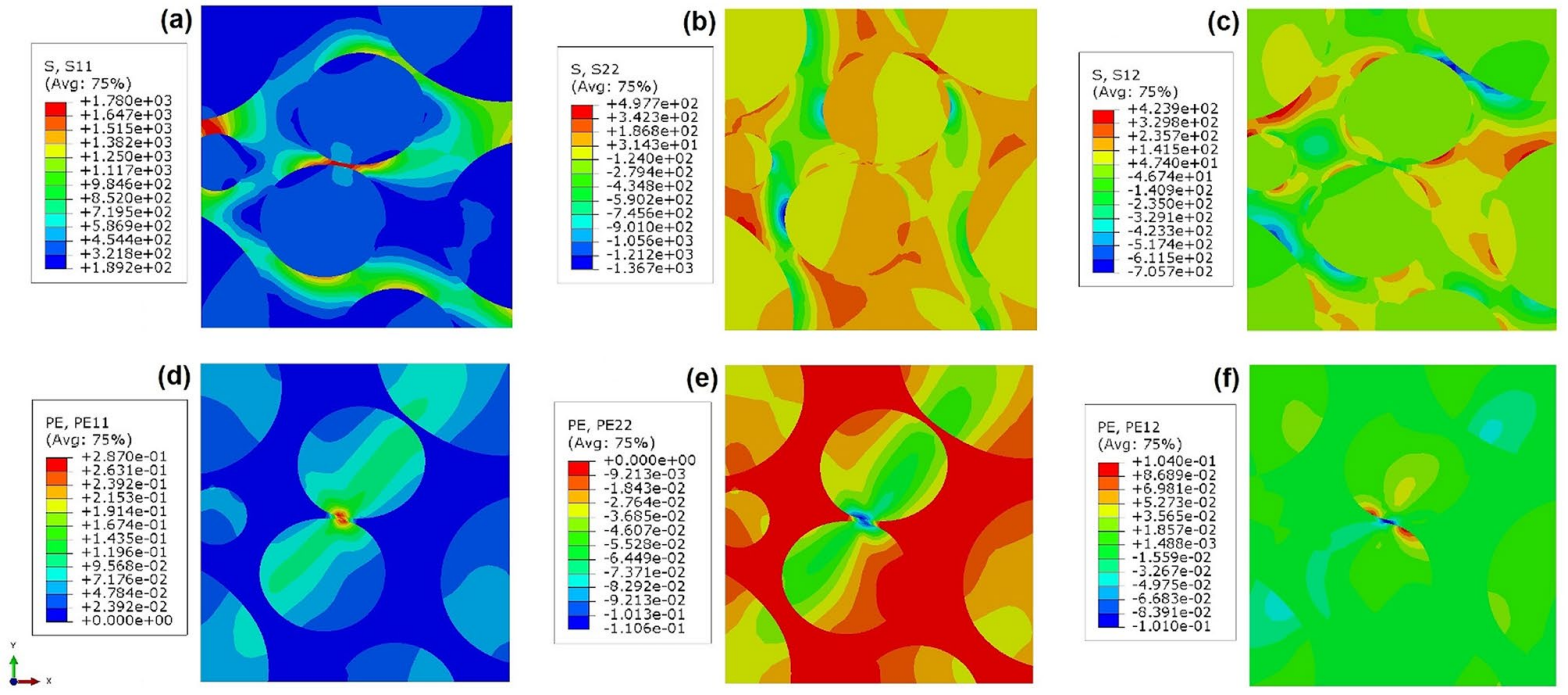
Distribution of normal *x*, *y*, and shear *xy* stresses (**a**–**c**) and plastic strain (**d**–**f**) tensor components at a global strain ${{\varvec{\varepsilon}}}_{{\varvec{e}}{\varvec{f}}{\varvec{f}}}=$ 0.04 in representative cells of bR material.

Figure 5 shows similar contour plots for the beginning of strain localisation in the bR sample at global strain ${{\varvec{\varepsilon}}}_{{\varvec{e}}{\varvec{f}}{\varvec{f}}}$ **=** 0.04. In general, the distributions of stress and strain tensor components tend to be somewhat similar to those in the bHS material. The extreme values can be found in the vicinity of UFG ‘necks’: highest stresses concentrate in UFG regions while highest strains in CG areas around them. However, the irregular distribution of CG/UFG phases leads to extremely heterogeneous irregular global stress/strain fields. Extended bands of high orthogonal strain components propagating across several CG and UFG areas form early and become intercepted by high shear strain strips within thinnest UFG regions. In turn, this causes premature concentration of equivalent strain to a critical level at global strain significantly lower than that for bHS material. These observations appear to be in a very good correspondence with the other results discussed below.

## Discussion

First, we compare stress–strain behaviour and analyse the components of stress–strain tensors along with macroscopic strain concentration patterns in both bHS and bR heterogeneous materials at the beginning of failure. Afterwards, we discuss these in comparison with other simulations and experimental results in the literature as well as our own earlier studies.

**Figure 6:**
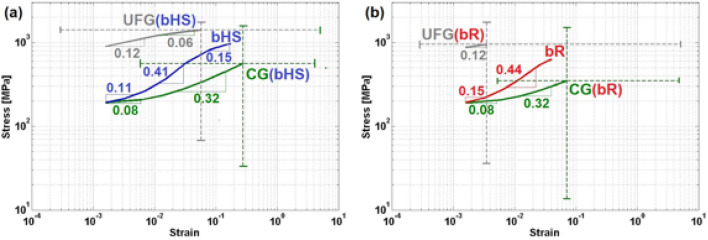
Mean true stress ${\sigma }_{eff}$ vs. true strain ${\varepsilon }_{eff}$ curves in log–log scale for the simulated (**a**) bHS and (**b**) bR materials. The curves represent the computed stress–strain dependencies separately for each CG and UFG phase and the entire material volume, while dash-line bars show ranges of stress and strain at ultimate tensile strength (initiation of strain localisation). The numerical values indicate strain hardening coefficients for each material and phases within at different stages of plastic deformation.

To better understand the reasons for the different behaviour of bHS and bR models, their stress–strain curves are presented in log–log scale diagrams in Fig. 6a,b, respectively. In these diagrams, the ‘master’ curves are reproduced along with separate curves for each phase, and strain hardening rates (inclination angles) are indicated for each linear section of the curves. The ranges of stresses and strains in each phase at full load of the master curve are also shown with dashed lines.

It is interesting to note that in the bHS material, Fig. 6a, (i) the ranges of stress–strain (shown by dash-lines) in CG and UFG phases overlap outlining a closed almost perfectly square area, and (ii) maximum stress–strain values in the master curve is almost in the centre of this area. By contrast, in the bR material, Fig. 6b, such area (i) is shifted towards lower stresses and strains also significantly expanding ranges towards lower values on both the axes, (ii) is not closed, and (iii) maximum strain in the master curve is shifted towards CG phase maximum. Furthermore, the maximum values of stresses and accumulated strains in each phase of bR material are smaller while variation ranges expand towards lower values compared to those in bHS one. Such variations suggest significantly more heterogeneous deformation in bR material, which leads to excessive local straining at early stages of strain concentration. It also leads to premature failure even though this is not given per se. This is noteworthy, considering that events such as motion of dislocations, crack growth, formation of traversing localised strain, etc. require homogeneous actions along their entire stretching but are often obstructed by irregular material properties.

When it comes to strain hardening, it can be seen that the UFG phase in both the materials has the same low rates of 0.12, which is constant along the entire curve in bR while changes to 0.06 at the later deformation stages in bHS. The CG phase also has similar trends but two strain hardening rates in both the materials. These are slightly lower than in the UFG phase at early stages, 0.08, and significantly higher, 0.32, at the developed stages of plastic deformation. The ‘master’ curve of the bHS material, Fig. 6a, reveals three strain hardening stages with rate coefficients 0.11, 0.41 and 0.15 at early, developed, and late stages of plastic deformation, respectively. It is interesting to find that the ‘master’ curve of the bR material, Fig. 6b, has only two strain hardening stages with rate coefficients 0.15 and 0.44.

**Figure 7:**
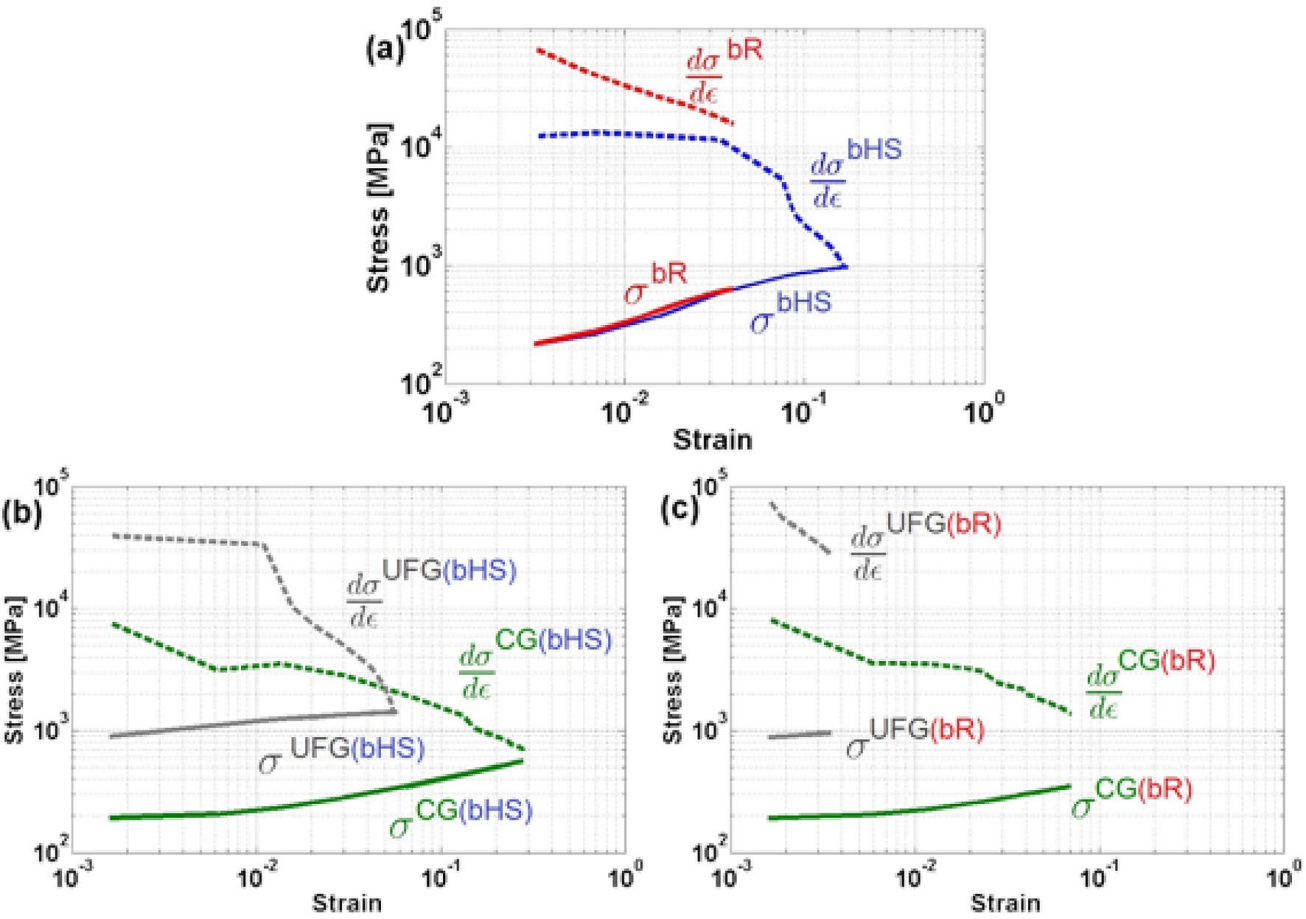
Mean true stress ${\sigma }_{eff}$ (solid lines) and strain hardening rate $\partial \sigma /\partial \varepsilon$ (dash lines) versus true strain ${\varepsilon }_{eff}$ curves in log–log scale for the simulated (**a**) entire volumes of bHS and bR material materials, and separately for each CG and UFG phase in (**b**) bHS and (**c**) bR materials.

Comparing strain hardening in the micro-architected materials with that in each phase, see Fig. 7, it can be said that the presence of second phase, or microstructure heterogeneity, always leads to higher strain hardening rates. The behaviour, or rheology, of bimodal materials is controlled by the CG phase at the early stages of plastic deformation and is strongly influenced by the UFG at the latest. Intermediate, i.e. developed, stage of plastic deformation is controlled by the compatibility of strain in both the phases. Nearly brittle fracture in randomly scattered UFG phase regions in the bR material, Fig. 7c, leads to premature fracture of the entire sample, Fig. 7a. By contrast, regular periodic distribution of the same phase in bHS material moderates strain gradients allowing extended compatible deformation of both the phases, Fig. 7b. This leads to the satisfaction of Considère's criterion $\partial \sigma /\partial \varepsilon =\sigma$ for strain localisation in both UFG and CG phases, Fig. 7b, as well as the entire sample of bHS material, Fig. 7a. Therefore, the design of bimodal material microstructure becomes critical in ensuring the compatibility of deformation. Less than optimal design in bR material leads to the premature failure, so that the ‘late’ stages of deformation cannot be reached. However, appropriate design of microstructure leads to the synergetic effect, as can be found in the bHS material.

**Figure 8:**
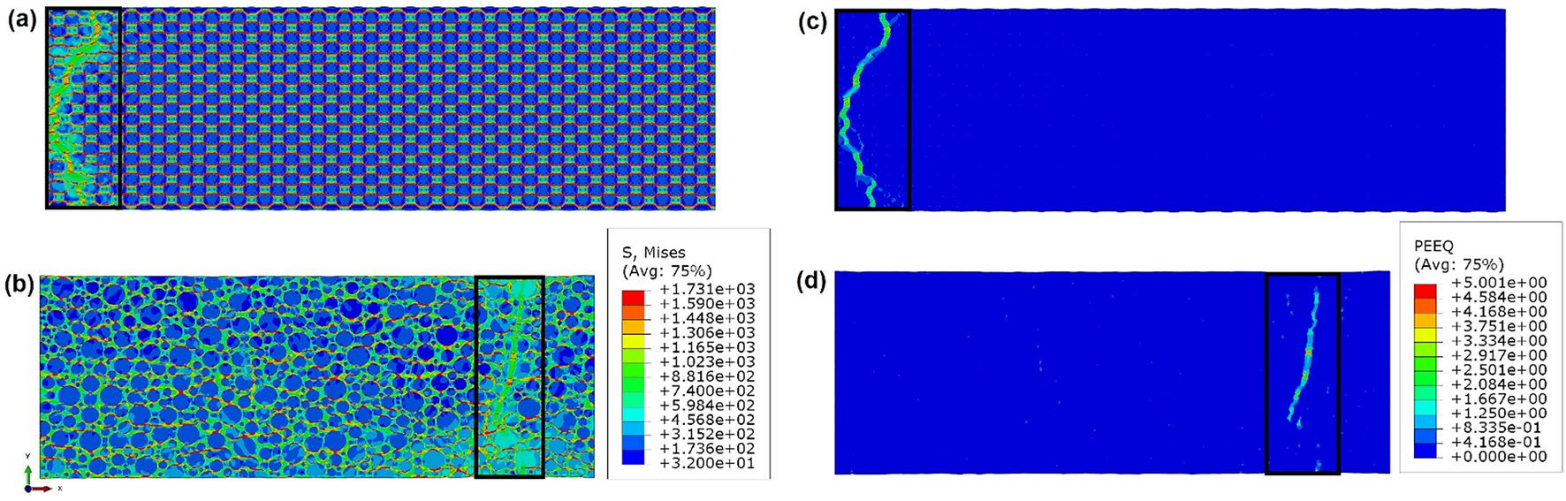
Distribution of von Mises stresses (**a**,**b**) and plastic strains (**c**,**d**) at a global strain ${\varepsilon }_{eff}=$ 0.2 in bHS (**a**,**c**) and bR (**b**,**d**) materials. The areas of ultimate plastic instability outlined by black squares are shown in greater details at various global strain levels in Fig. 9.

Figure 8 shows contour plots for von Mises stresses and effective plastic strains at the beginning of plastic instability (start of global strain localisation, or the start of catastrophically rapid FE distortions in these simulations). Note that due to the limitations of our model, we consider only the beginning of plastic instability, while the process of severe strain concentrations is ought to be considered for future studies. It can be seen that the concentrations of plastic strain within specimens and paths are very different between bHS than bR materials.

The path of the globally localising strain in bHS sample is quite torturous with alternating direction of propagation across the entire specimen cross-section. Such properties of failure propagation are typical for fracture in materials with reasonably high ductility. The symmetry boundary condition in the centre of our model might affect the process of plastic instability formation, as can be guessed from the field of elevated von Mises stresses interfering with the symmetry axis in Fig. 8a. However, such effects are expected to be negligibly small not altering final results since the high-strain path never comes close to the symmetry axis on the left, see Fig. 8c. Even if the proximity of strain concentration to the axis of symmetry would play any sensible role, this would lead to a lower global plastic strain until strain localisation initiates in the bHS sample.

The path of strain concentration in bR sample is principally different, see Fig. 8d. It is unidirectional, rather straight, and almost perpendicular to the tensile axis. Such properties of fracture in tensile testing are typical signatures of brittle failure.

The described differences can be associated with more uniform (regular) distribution of CG/UFG areas in the bHS sample leading to the delay of strain localisation.

**Figure 9:**
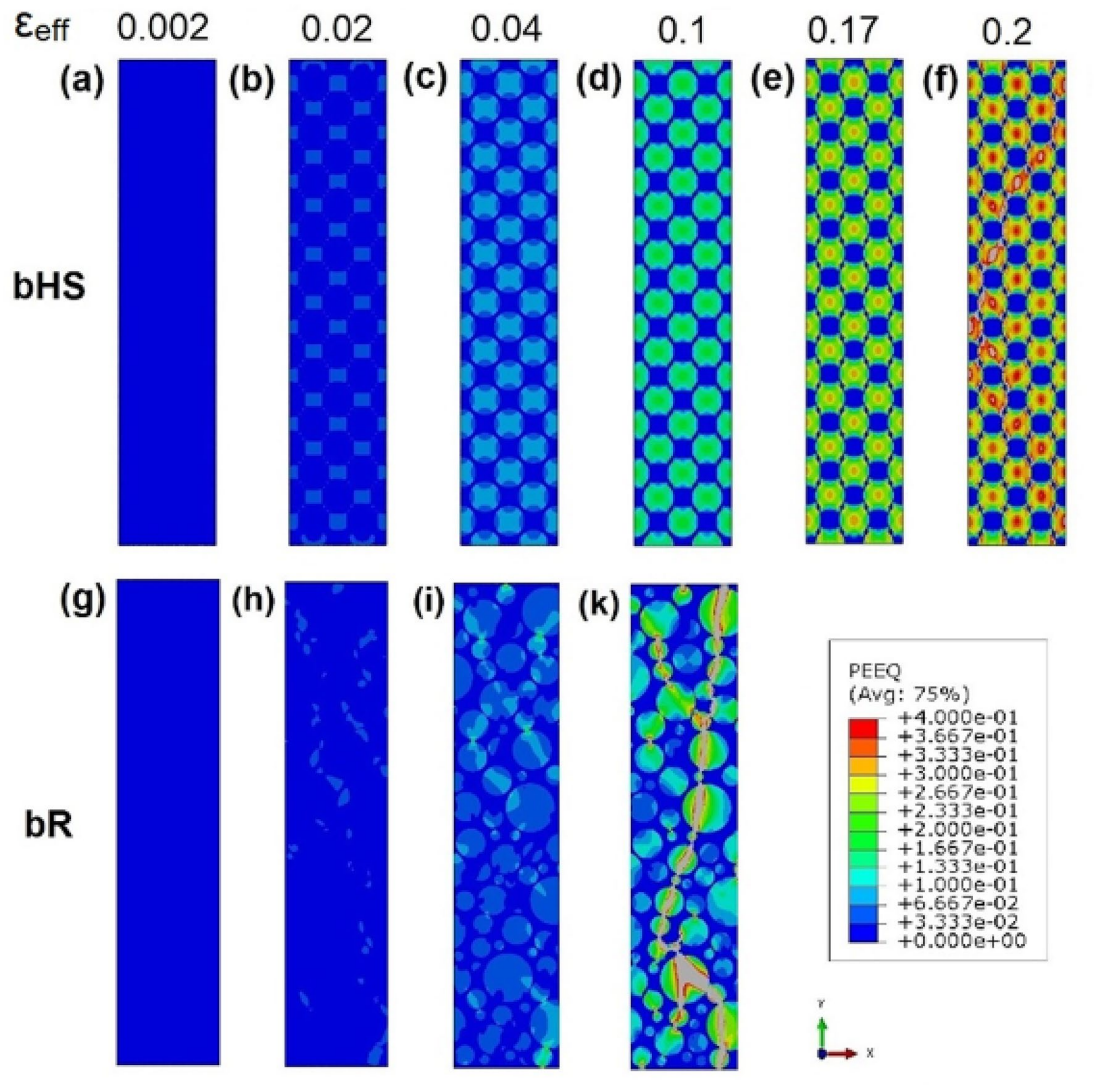
Distribution of plastic strains in selected areas around global strain localisation in bHS (**a**–**f**) and bR (**g**–**k**) specimens at various global plastic strain increments ${\varepsilon }_{eff}$ indicated at the top row.

Deeper analysis of strain concentration pattern in the bHS and bR samples is carried out in Fig. 9 where the specimen areas of strain concentration at representative global strain increments are presented. Last two increments in each series, Fig. 9a–f for bHS and Fig. 9g–k for bR, show equivalent plastic strain distributions immediately before and after catastrophic local strain increase.

In the bHS material, plastic strain starts in the middle and then propagates along maximum shear stresses in ± 45° directions with regard to the applied load until CG volumes become plastic almost entirely, see Fig. 9a–e. Up until this stage the strain distribution is still homogeneous globally, as described in detail in Supplementary information S1. The transition between Fig. 9e,f can be characterised by the sharp increase of strain concentration values in the middle of the CG areas and their interfaces with UFG skeleton ‘necks’ in ± 45° directions. These ultimately lead to global plastic strain instability at the specimen-scale predominantly propagating through the described areas at ± 45° angles.

In the bR material, plastic strain distribution is heterogeneous globally from the moment it starts, as described in detail in Supplementary information S1. Strain concentration increases first at the periphery and then propagates into the volume of CG areas, see Fig. 9h, i. The angle of strain propagation often deviates from the maximum global shear stress directions of ± 45° with regard to the applied load because of local stress field variations. As revealed by Fig. 9k, maximum local strains after global plastic instability propagate through the network of CG areas making shortest path through UFG regions. Such a path formation is apparently incidental and premature, which is the consequence of a random mutual distribution of CG and UFG phases.

For both bHS and bR materials, the ends of the modelled part are given symmetry boundary conditions which means that the thinning of the specimen in the *y*-direction is shared, so that only reduced part of necessary work required for the progression of rupture. The available energy is at the necking moment supplied by the unloading remain part for the specimen. The localisation in the vicinity of an end means that the available free elastic energy is required to support more or less half of the zone of strain localisation. Localisation solitary zone distanced from the ends require support for the entire localisation zone as a whole. This favours localisation at the ends.

The upper and the lower surfaces at *y* = 0 and 1 mm tractions are absent. Inside the specimen, say at 0.1 < *y* < 0.9 mm there is tensile vertical stress in the CG and compressive ditto in the UFG material. Both materials have tensile stress in the *x*-direction, which means plastic deformation in the *x* direction is promoted in the UFG material and inhibited in the CG material as compared with the mechanical state of the surface. This especially obvious for the bR model where the local concentration starts in the interior of the specimen, cf. Fig. 8b. The differences between surface and interior regions lead to a stable initiation of the developing localisation, which promotes localisation at the ends with a faster relaxation of the remaining parts and increases the load at the ligament like adjacent surface.

In bR case, the statistical variation provides additional opportunities for strain localisation that in the present study, at least in one place, increase the energy release rate, so that it exceeds what would be gained by localisation at any of the two ends. The outcome of strain localisation traversing an inner cross section of the modelled part, results in two necking regions per 5 mm, as can be imagined by fully unfolding the symmetry conditions.

The results presented in this work are in a very good agreement with reports from the literature. For instance, with qualitative evolution of strain distributions in a 3D model^36^ closely resembling HS material. Our results are also consistent down to quantitative level with works^37,38^ where authors developed a 3D multi-scale polycrystalline plasticity model coupled with damage and applied it to study the grain size effect on the macroscopic inelastic behaviour in commercial-purity polycrystalline Ti with HS. In particular, the concentration of strain in CG areas upon global strain increase from 0.00346 to 0.00693 in^38^ agrees well with our results presented in Figs. S1.6a–c. The added value of our work is that it does not only explain the behaviour of bimodal harmonic-structure material but also reveals its difference from a counterpart with random spatial distribution (topology) of CG and UFG phases. Moreover, it also demonstrates that bHS design provides synergetic effect significantly exceeding expectations from the rule of mixtures, especially for material ductility. In turn, this emphasises the importance of topological UFG–CG phases distribution, and the continuity of UFG skeleton in particular.

Our results agree well, also with experimental reports. For instance, Park et al^40^ studied HS stainless steel SUS304L and demonstrated using micro-digital image correlation analysis that local strains are concentrated in CG areas around core/shell interfaces already at $\epsilon =0.12$. Studying Al–Mg alloys with bR grain size distribution, Fan et al^41^ experimentally showed that at similar global strain of $\epsilon =0.12$, local strains are concentrated in the CG areas forming shear bands along approximately 45° to the direction of loading. Similar results are discussed in this work earlier. Comparing present results with our earlier experimental work on pure Ni^24^, one can see that the FE models do reproduce the trends qualitatively but substantially underestimate maximum stress–strain values achieved in heterogenous bimodal materials at UTS. Such a discrepancy can be attributed the lack of a material fracture component in the present FE model. The development of the FE model by introducing more realistic UFG-CG areas morphology from real-life micrographs segmentation along with improving material rheology, and introducing fracture component in particular, will be prime and immediate focus in follow-up studies.

## Conclusions

In this work, 2D finite-element (FE) models for bHS (bimodal harmonic-structure) and bR (bimodal random) distributions of grain sizes are developed. These models allow the numerical investigation of elastic–plastic behaviour in respective heterogenous materials for clarifying the evolution of distributions in stresses, strains and structural components/phases. The models are then used for investigating the aforementioned material characteristics in uniaxial tension on the example of pure nickel. From the analysis and comparison of results, phenomenological mechanisms of plastic deformation in materials having heterogeneous grain structure are clarified, while reasons behind the importance of topological UFG/CG distributions and the continuity of the UFG skeleton in particular are revealed.

In consistence with other reports, the bHS material demonstrates regular (homogeneous) stress–strain distributions at macro-scale along with their significant local variations. While the former remains homogeneous to relatively high global strain levels, the later significantly increases in amplitude and gradient around the centre of soft CG areas and their interfaces with hard continuous UFG skeleton. Such strain partitioning leads to strain hardening rates significantly exceeding those in homogeneous materials. The strain hardening rates are primarily controlled by CG phase at the low plastic strains and by UFG at the larger straining. At the intermediate stages of loading, the compatibility of plastic flow, i.e. strain partitioning between the increasingly constraint CG phase and the hardened-to-saturation UFG skeleton, plays a key role in the composite-like material strengthening behaviour. The bHS design provides synergetic effect significantly exceeding expectations from the rule of mixtures, especially for material ductility.

The bR material develops heterogeneities in stress–strain distributions at all scale levels (i.e., the lack of macro-scale homogeneity) immediately upon loading. This leads to rapidly developing strain concentrations and premature global plastic instability. These are reflected in strain hardening rates higher than in the bHS material while showing two stages of deformation only.

Although developed models fully capture and explain qualitatively the experimental results, further development of the models is necessary for quantitative predictions. Such a development will be reported in follow-up publications based on ongoing studies.

## Methods of computational numerical model for simulations

### General considerations

The connection between model materials to be used in the present study and real-life microstructures is presented in Fig. 1. HS materials are typically fabricated from coarse-grained spherical powder particles approximately 100–200 μm in diameter^21^. In the prototype experimental study^24^, 150 μm diameter particles were used, and the morphology of CG areas is broadly preserved in the bHS material in Fig. 1c. It can also be found from the figure that CG areas are approximately 120 μm in average diameter crossing up to 10 grains in one direction. Three-dimensional consideration of such areas suggests the number of grains in each at the order of 200. Similar geometric considerations can be applied to the UFG phase forming intra-circular skeleton, as depicted by dark-grey colour in Fig. 1c. The 40% UFG fraction suggests ligaments of 10–40 μm consisting of grains with sub-micron diameters, which puts the number of grains in shortest skeleton sections at the order of several hundreds and orders of magnitude more in other areas.

Furthermore, since sample fabrication route involves high-temperature sintering of randomly-oriented powder particles^24^, such a large number of virtually randomly-oriented grains in high stacking-fault energy cubic nickel suggests the material to be texture-free. Although all crystalline materials including metals have anisotropic elastic and plastic properties, these are averaged out in polycrystals if the number of grains is sufficiently large, and the orientations are random. Since this is the case in our study, crystallographic orientations and anisotropy of grains can be neglected, and the materials of different rheology in each phase can be simulated in the model as homogenous isotropic elastic–plastic.

It is appreciated that the specific crystallographic orientations of near-interface grains do play a role in the local strain partitioning, e.g. the variation of stresses and accumulated plastic strains in the CG-UFG interface regions, but they do not exceed 10% of the averaged values, as was demonstrated experimentally in^40^ and analytically in^16^. Additional arguments for intentional model simplifications by excluding the effect of grain orientations refer to our aim in this work to focus primarily at meso-scale effects of elastic–plastic behaviour in bHS and bR materials. Consideration of the former would make the model virtually unsolvable since thousands of grains would have to be considered even at a smallest representative volume element scale, as explained above.

Based on the aforementioned material fabrication route and the microstructure characterisation results in Fig. 1, CG areas can be considered circular in the FE model. The 3D model of HS material assumes to resemble atomic packing in face-centred cubic crystal structure, which is known to be the most densely packed of all homogeneous structures. This allows further simplification of our model making it consisting of repeating CG spherical areas embedded into the skeleton of UFG in all the three dimensions of the Cartesian space. Namely, the substitution of periodicity in one dimension, i.e. small sample thickness in *z* direction compared to length and width in *x* and *y* directions, respectively, can be compensated in the two-dimensional (2D) ‘plane stress’ model by the sufficient number of unit cells to achieve macroscopically homogeneous structure. Earlier results in our group^42^ revealed that a 2D FE model of a tensile test sample with harmonic structure using plane-stress elements is preferred over its 3D counterpart since it allows better spatial resolution in the areas of interest (CG-UFG interfaces) along with the reduction or at least the same computational cost. A 2D ‘plane strain’ model would mean the consideration of grains as columnar extruded in the thickness direction, which cannot be applied to our case. A ‘generalised plane strain’ model, which permits off-plane 2D element distortion, gives solutions very close to those in ‘plane stress’ model, at least in the range of strains reported in this study. 2D axisymmetric model was also ruled out since benchmark uniaxial tensile tests^24^ (from which source experimental stress–strain curves were utilised) were carried out on flat dog-bone shaped specimens. More detailed considerations about the selection of a specific model for simulation in this study and the accuracy of calculations are presented in Supplementary information S2.

Some recent studies suggest the advantages of 3D crystal plasticity models over simpler 2D alternatives. Interestingly enough, the conclusions from these studies are consistent with our own investigation in Supplementary information S2 and in fact can support our selection of the most appropriate model. In^43^, the effect of grain morphology on strain distribution at a free surface in polycrystalline aggregates was investigated. The number of grains considered in the model was relatively low, 39, and the 3D model itself was further limited to coarse grains only. In^44^, “real 3D microstructures” of dual-phase steels were studied for stress and strain partitioning between phases upon mechanical loading. Although the number of grains in the simulated volume was not given explicitly, it can be estimated as up to 55 “coarse” ferrite grains along with 20 “ultrafine” martensite grains. In both the papers, the FE simulations in 3D models are benchmarked against ‘2D columnar extruded grain’ models that are effectively 2D ‘plane strain’ models. Similar to our evaluation, 2D ‘plane strain’ models do not adequately represent 3D morphology of the structures under consideration and therefore overestimate local stresses and strains. By contrast, 2D ‘plane stress’ model is adequate for the present study, and we decide to stay with it.

We do understand that such simplifications are significant, especially considering modern state-of-the-art in FE-based crystal-plasticity simulations. However, a large number of experimental works on HS materials and almost negligible number of theoretical and computational models for simulating such along with enormous interest in improving structural efficiency of gradient-structure metallic materials, motivates this study. We expect it not to be exhaustive but rather to answer the most acute conceptual questions of today in the area, to identify boundary conditions for studying localised effects that are important and are thus worthy separate investigations using more sophisticated specialised computational models.

### Material model

Both nickel phases are modelled as isotropic elastic–plastic with strain hardening based on uniaxial tensile test results of^24^ and^45^ for 100% CG and 100% UFG nickel, respectively. The elastic material properties follow Hooke's law and Poisson's ratio taken from tabulated material parameters for pure nickel, i.e., the modulus of elasticity ${E}_{CG}=210$ GPa and Poisson’s ratio $\nu =0.31$, cf.^42,45^. According to the plastic stress–strain data in^24^ and^45^, engineering yield stresses of the CG and the UFG phases are taken as 185 MPa and 1502 MPa, respectively. The UFG Ni in as high-pressure torsion processed state at an accumulated equivalent strain of *e* = 55^45^ is considered as a saturated strain hardened condition. Therefore, the stress–strain dependence beyond ultimate tensile strength is considered to be constant.

For the numerical calculations, von Mises yield criterion is used to determine the onset of plasticity and the plastic deformation. The latter is given by the associated flow rule.

At the tensile testing of the CG and the UFG specimens before maximum load and subsequent strain localisation, the stress is uniaxial and homogeneous along the entire gage length. The engineering tensile stress is denoted ${\sigma }_{exp}$ and the engineering strain is ${\epsilon }_{exp}$. For a homogeneous specimen with a constant cross section, the strain, ${\epsilon }_{t}$, in the plane perpendicular to the tensile direction is isotropic, i.e. independent of direction.

### Conversion of the tensile test data

The FE calculations require the tensile data to be input as true stress *vs* the true plastic strain.

The Cartesian coordinates *x*, *y* and *z* are used with *x* in the tensile direction along the specimen long axis, while *y* and *z* are aligned with the other orthogonal directions along the sides of rectangular cross section, *y* in-plane and *z* out-of-plane.

The strain components are split into a linear elastic part given by the Hooke's law and a plastic part for which the volume preservation condition is fulfilled. This provides the following definitions of the engineering plastic strain components:

2 $$ {\epsilon }_{exp}^{p} = {\epsilon }_{exp} - \frac{1}{E}\sigma_{exp} \;{\text{and}}\; {\epsilon }_{t}^{p} = {\epsilon }_{t} + \frac{\nu }{E}\sigma_{exp} . $$

Engineering quantities relate to the original cross section area $A_{o}$ and gage length $\ell_{o}$ in its undeformed state, whereas the true quantities relate to the actual instantaneous area $A$ and length $\ell$. We observe that the plastic strain does not affect the volume and by recognising that the elastic strains are very small and also almost instantly after initiation of plastic deformation become insignificant beside its plastic counterparts. Preservation of the volume $\ell t^{2} = \ell_{o} t_{o}^{2} \Rightarrow A/A_{o} = (t/t_{o} )^{2}$ leads to the following relation between engineering and true stress:

3 $${\sigma }_{x}={\sigma }_{exp}(1+{\epsilon }_{\mathrm{exp}}).$$

The relations (2) and (3) are used to convert the engineering tensile data of^24^ and^45^. The force displacement relations are converted to von Mises effective true stress $\sigma$ as a function of the engineering effective plastic strain $\epsilon$.

After maximum load and strain localisation, the conversion loses its accuracy whereas the global strain strongly overestimates load carrying capacity of the segment where the strain localises. A crude estimate could be obtained by downscaling the strain added after localisation is initialised with a factor of gage length versus the thinnest side of the rectangular cross section. The method is slightly speculative, and the result is uncertain due to an uncertainty as regards the localisation process. Therefore, no adoption of the result to the strain localisation situation is performed.

**Figure 10:**
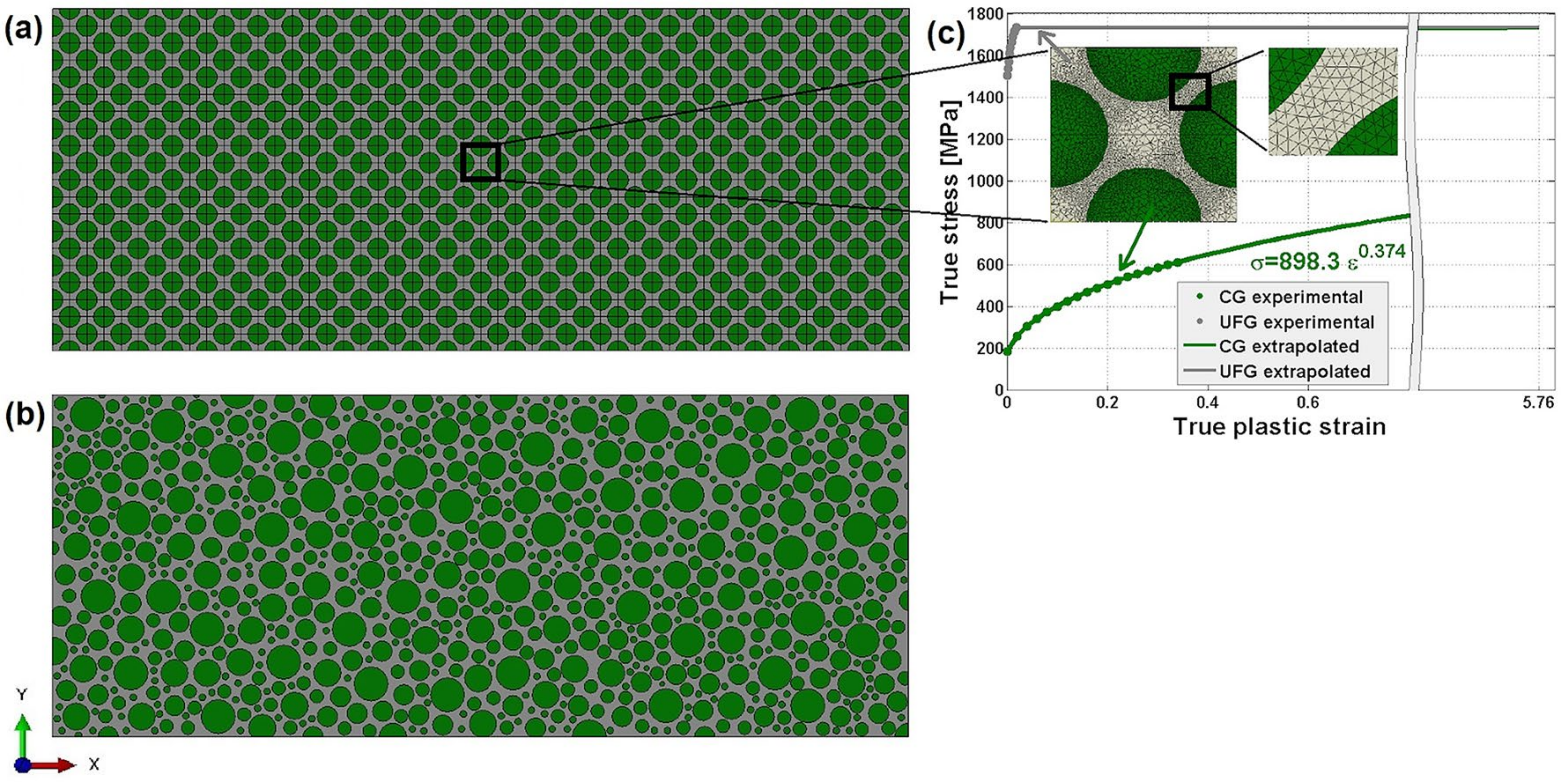
2D models for (**a**) bHS and (**b**) bR samples where green and grey areas represent CG and UFG structure phases, respectively; (**c**) tensile curves for the effective true stress versus true plastic strain used for describing the rheology of CG and UFG phases in the simulations. Experimental stress–strain data are shown by dots, while Ludwig-Hollomon relationship fit by solid lines. Insets in (**c**) show magnified views of the meshed bHS unit cell.

The true stress versus true strain curves used for the simulations are presented in Fig. 10c. For the coarse-grained phase, the plastic part of experimental stress–strain data is extrapolated by a power law using the Ludwik-Hollomon relationship^46^ $\sigma =898.3{\epsilon }^{0.374}$, with the definitions of effective stress and strain as following

$\sigma =\frac{1}{\sqrt{2}}\sqrt{({\sigma }_{2}-{\sigma }_{3}{)}^{2}+({\sigma }_{1}-{\sigma }_{3}{)}^{2}+({\sigma }_{1}-{\sigma }_{2}{)}^{2}}$and $\epsilon =(\sqrt{2}/3)\int \sqrt{({\text{d}}{\epsilon }_{2}^{p}-{\text{d}}{\epsilon }_{3}^{p}{)}^{2}+({\text{d}}{\epsilon }_{1}^{p}-{\text{d}}{\epsilon }_{3}^{p}{)}^{2}+({\text{d}}{\epsilon }_{1}^{p}-{\text{d}}{\epsilon }_{2}^{p}{)}^{2}}$where the stresses and strains ${\sigma }_{1}^{p},{\sigma }_{2}^{p}...{\epsilon }_{3}^{p}$ are the principal true stresses and the principal plastic engineering strains.

The notation $\sigma$ is for the von Mises effective true stress and $\epsilon$ is for the effective engineering plastic strain. The Fig. 10c shows the stress $\sigma$ versus strains $\epsilon$ as they are obtained from the experimental data.

### Finite element model

An updated Lagrangian scheme is used to keep track of large deformations in the *x*–*y* plane, cf.^47^. The material model for the two-dimensional FE simulations is based on our earlier experiments^24^ replicating the most critical parameters. The simulations focus on commercial purity nickel (Ni, ≥ 99.2%) powders with an average particle size of up to 120 μm. Further details about fabrication procedures for the material can be found in^24,25^. The UFG fraction in both bHS and bR models is selected to be 40%, which is based on previous studies demonstrating the best structural characteristics in harmonic-structured nickel, copper, and stainless steel SUS304L^24,25,48,49^. Dimensions of the simulated specimen are length × width × thickness = 5 × 1 × *h* mm^3^, where the thickness *h* is arbitrary but supposed to be sufficiently less than 0.1 μm to secure plane-stress conditions. The periodic structure including a unit cell geometry is shown in Fig. 10. The initial inter-particle distance is 0.1 mm.

Periodic unit cell to simulate 1/200th of the specimen, a strip stretching from the top surface to the specimen mid-plane or a four-folded symmetry conditions, quarter of the specimen gage would be routinely used. For specimens with smaller thickness than width, asymmetric deformation modes and subsequent strain localisation are expected after peak load. In our case, it is interesting to study the beginning of localisation and the degree of asymmetry in comparison with known results for strain localisation in homogeneous materials.

Simulating the full-length specimen gage would demand an unreasonably long calculation time. An alternative is using a coarser element mesh, but this leads to a reduction in the resolution that may be detrimental to the analysis of the expected strain concentrations. This leaves us with the remaining alternative to model only half of the specimen length, i.e. for a specimen gage of 5.0 × 1.0 mm^2^, the treated part becomes 2.5 × 1.0 mm^2^. Thickness is arbitrary but restricted to being significantly smaller than the linear in-plane extent of a unit cell.

In Fig. 10a, b, symmetry boundary conditions in the x-direction are applied at the left and right sides, i.e. *x* = 0 and 2.5 mm, respectively. At *x* = 0, the centre point on the surface is prevented from moving in the *y*-direction for numerical reasons to prevent numerically induced loss of equilibrium. A uniform displacement, *u*_*x*_ = *u*_*o*_, is applied in the *x*-direction at the right surface (*x* = 2.5 mm and 0 ≤ *y* ≤ 1 mm), for both models. The left side (*x* = 0 and 0 ≤ *y* ≤ 1 mm), is constrained to *u*_*x*_ = 0. Shear tractions *σ*_*xy*_ = 0 on both left and right sides. The upper and the lower surfaces at 0 < *x* < 2.5 mm, *y* = 0 and 1 mm are traction-free, ${\sigma }_{y}$=${\tau }_{xy}$=0. For the remaining sides parallel to the *x–y* plane, plane stress conditions ${\sigma }_{z}\hspace{0.17em}=\hspace{0.17em}{\tau }_{xz}\hspace{0.17em}=\hspace{0.17em}{\tau }_{yz}$  = 0 are assumed.

Two 2D FE models are developed for the simulations in Abaqus/CAE^47^. Figure 10a shows the first model for the bHS sample with a periodic structure of repeated unit cells, as outlined by a black rectangle and shown in magnified-view insets for clarity. The second model is for the bR sample with a heterogeneous structure of randomly distributed both CG area sizes and their spatial positioning within UFG matrix, as shown in Fig. 10b.

The development of stress and strain gradients around UFG area of the thinnest cross-section between CG areas has a particular interest because of their role as design-dictated ‘necks’. Therefore, the dimensions of the finite element density are selected to make at least 10 nodes (8 elements) across such sections. The details of meshing in the vicinity of such areas are illustrated in the insets of Fig. 10c. They show the meshing of a bHS cell with 5254 plane-stress three-node constant-strain elements, type CPS3 in Abaqus notation^47^. The total number of elements is 1,370,706 and 1,038,891 for the bHS and bR models, respectively.

## Supplementary Information

Supplementary Information 1.

Supplementary Information 2.
