# Supplementary material for: Improvement of structural efficiency in metals by the control of topological arrangements in ultrafine and coarse grains
Source: Sci Rep. 2021 Aug 31;11:17445. doi: 10.1038/s41598-021-96930-3 (PMC8408239; doi:10.1038/s41598-021-96930-3)
Supplement: Supplementary file 1 — Supplementary Information 1. [file 41598_2021_96930_MOESM1_ESM.docx]

**Improvement of structural efficiency in metals
by the control of topological arrangements in ultrafine and coarse grains**

***Authors:*** Abdallah Shokry, Aylin Ahadi, Per Ståhle, Dmytro Orlov*
*Corresponding author, email: dmytro.orlov@material.lth.se.

**Supplementary information S1**

**In-depth analysis of spatial stress and strain distributions in bimodal random (bR) and bimodal harmonic-structure (bHS) materials at five characteristic strain levels**

***Stress-strain distributions at elastic-plastic transition,*** $\boldsymbol{\varepsilon}_{\boldsymbol{eff}}$ ***= 0.002***

The distribution of von Mises stress at accumulated global strain $\boldsymbol{\varepsilon}_{\boldsymbol{eff}}=$0.002 is presented in **Fig. S1.1** for all the simulated materials. Purely CG (**Fig. S1.1a**) and UFG (**Fig. S1.1b**) models reveal very homogeneous von Mises stress fields at the levels of 180MPa and 70MPa, respectively. These suggest that UFG material experiences purely elastic strain, while the CG one is very close to plastic yielding already.


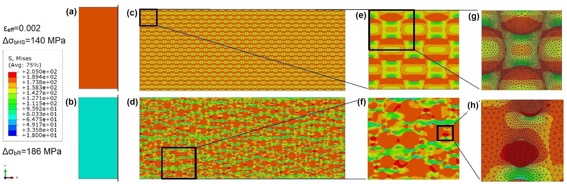


**Fig. S1.1**. Distribution of von Mises stresses at a global strain $\varepsilon_{eff} =$0.002 in (a) 100% CG, (b) 100% UFG, (c, e, g) bHS, and (d, f, h) bR materials; (e-h) show magnified areas outlined by respective black squares at lower-magnification distributions; FE meshes in deformed shapes are shown in (g, h) for the ease of CG / UFG phase identification. Colour codes on all the images correspond to a scale bar shown on the left-hand side along with reference directions and ranges between max-min stress values in each bimodal material.

**Figs. S1.1c**, **e** and **g** reveal significant local variation of stresses at a unit cell scale along with their regular macroscopic distribution at the specimen-scale in the bHS material. It can be seen that stresses are lower in the centre of CG areas and increase to a highest level towards the periphery / interfaces with the UFG skeleton. The stress gradients are higher towards the top / bottom surfaces of CG areas, i.e. in the ± direction normal to applied load. In the UFG skeleton regions the stress gradients are inverted, i.e. stresses are higher in their centres and decrease towards interfaces with the CG regions. The lowest von Mises stress levels can be found at the top / bottom regions of UFG skeleton at the interface with the CG phase (i.e. ± direction normal to applied load from the centre) while the highest are in the UFG ‘neck’ regions between two CG areas, i.e. inclined for approximately ±45° from the regions centre, see **Figs. S1.1e** and **g**. An additional local effect of an excess stress can be seen at the first sub-surface layer in depth of both top and bottom sides of the sample. As can be analysed further from **Figs. S1.1e** and **g**, the excess stress is lower at the thinnest UFG ‘necks’ and higher towards the centres of CG areas in sub-surface unit cells. The range of stress variation in bHS model is Δσ_bHS_ = 140MPa, which indicates that the material is still elastic everywhere in the volume in spite of local rather modest stress gradients.

The bR material, see **Figs. S1.1d**, **f** and **h**, reveals no regular pattern in von Mises stress levels. The stresses are distributed randomly at both specimen and CG / UFG region scales. The only similarity with the bHS model is higher stress levels in the CG phase, which can be evenly distributed within respective areas when they are small enough, see **Figs. S1.1f** and **h**. The range of stress variation in bR material is $\Delta\sigma_{bR}=$186 MPa, which is more than the yield stress of the CG phase used in the model thus suggesting local plastic flow in high-stress CG areas.

The distribution of accumulated effective plastic strains in all the simulated materials is shown in **Fig. S1.2**. Both the homogeneous CG (**Fig. S1.2a**) and UFG (**Fig. S1.2b**) models reveal perfectly homogeneous accumulated strain fields at the levels of $\varepsilon_{eff}$*<*0.0009 and $\varepsilon_{eff}$*<*0.0003, respectively. Presuming the global threshold for plastic yielding of $\epsilon$ *=*0.002, these suggest that both the homogeneous materials deform in a purely elastic regime still.


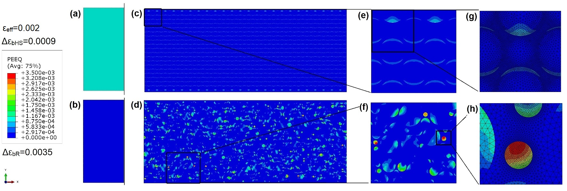


**Fig. S1.2** Distribution of accumulated effective plastic strains at a global strain $\boldsymbol{\varepsilon}_{\boldsymbol{eff}}=$0.002 in (a) 100% CG, (b) 100% UFG, (c, e, g) bHS, and (d, f, h) bR materials; (e-h) show magnified areas outlined by respective black squares at lower-magnification distributions; FE meshes in deformed shapes are shown in (g, h) for the ease of CG / UFG phase identification. Colour codes on all the images correspond to a scale bar shown on the left-hand side along with reference directions and ranges between max-min plastic strain values in each bimodal material.

**Figs. S1.2c**, **e** and **g** suggest that the plastic strain level is very homogeneous and low, $\varepsilon_{eff}$*<*0.0003, at the specimen-scale in the bHS material. At the same time, a unit cell scale plastic strain concentration spikes to $\varepsilon_{eff}$*<*0.0009 can be found at the top / bottom areas of the CG phase in immediate vicinity with the UFG skeleton. These spikes are very local, elongated in the direction of loading and regular macroscopically in the specimen volume. At the same time, additional strain fields slightly extended towards periphery in the direction normal to loading can be seen at the sub-surface layer of CG phase on both top and bottom sides of the specimen, see **Figs. S1.2e** and **g**. Note that all these strains are still below the adopted yield threshold $\epsilon$*=*0.002.

In the bR material, see **Figs. S1.2d**, **f** and **h**, accumulated effective plastic strains distribute extremely heterogeneously. While the majority of the specimen volume is still deformed elastically, some smallest-size CG areas already deform plastically at the level of $\varepsilon_{eff}$*=*0.0035.

***Stress-strain distributions at*** $\boldsymbol{\varepsilon}_{\boldsymbol{eff}}\boldsymbol{=}$***0.02 (strain concentration in 100% UFG material)***

At this global strain level, homogeneous 100% UFG material is in the state of plastic strain instability (localisation) already and is therefore excluded from following consideration. The simulations also demonstrate that the 100% CG material has homogeneous stress and plastic strain distributions at all global strain levels of interest in this study. This fully agrees with expectations from the stress-strain data, **Fig.10c**, and is therefore also excluded from following discussion for the sake of saving journal space.

**Figs. S1.3a**-**c** reveal further development of local stress gradients at a unit cell scale, while their macroscopic distribution at the specimen-scale remains regular in the bHS material. With the qualitative pattern of local stress gradient being preserved, its amplitude variation between CG and UFG areas becomes so large, $\Delta\sigma_{bHS}=$828MPa, that the stress variation within each phase appears homogeneous (i.e. single colour-scale range). The high-stress areas make a continuous wavy pattern undulating around the direction of loading through UFG regions. In perpendicular direction, narrow intermittent high-stress pattern propagating through the UFG phase adjacent to CG areas can also be seen. The mean von Mises stress level in the CG phase is at the level of 300MPa, while in the UFG phase it varies from 110MPa in the middle of skeleton nodes to 940MPa in the ‘neck’ regions. These suggest that the majority of CG phase is plastic already while UFG phase is still elastic.


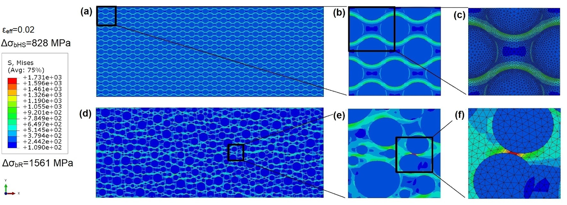


**Fig. S1.3** Distribution of von Mises stresses at a global strain $\boldsymbol{\varepsilon}_{\boldsymbol{eff}}=$0.02 in (a, b, c) bHS, and (d, e, f) bR materials; (b, c, e, f) show magnified areas outlined by respective black squares at lower-magnification distributions; FE meshes in deformed shapes are shown in (c, f) for the ease of CG / UFG phase identification. Colour codes on all the images correspond to a scale bar shown on the left-hand side along with reference directions and ranges between max-min stress values in each bimodal material.

The bR material, see **Figs. S1.3d**-**f**, again reveals no regular pattern in von Mises stress distribution. The range of stress variation increases to $\Delta\sigma_{bR}=$1561 MPa. High-stress regions can be found randomly distributed predominantly in UFG regions, while stress gradients are visible even in CG areas. The highest levels of stress in excess of 1700 MPa can be found in narrow UFG ‘necks’ between CG areas, while the level of stress in the latter occasionally exceeds 380 MPa. These suggest that both CG and UFG phases are plastic in bR material at least in some regions.

**Figs. S1.4a**-**c** suggest that in the bHS material the plastic strain gradients at this global strain level further evolve similar to the stress distribution pattern. In the UFG skeleton plastic strains appear homogeneous and never exceeding the elastic threshold of $\epsilon$*=*0.002. The CG areas appear to be fully plastic with accumulated plastic strains varying from $\boldsymbol{\varepsilon}_{\boldsymbol{eff}}$ < 0.04 in the majority of interior down to $\boldsymbol{\varepsilon}_{\boldsymbol{eff}}$ < 0.016 at their top / bottom tips.


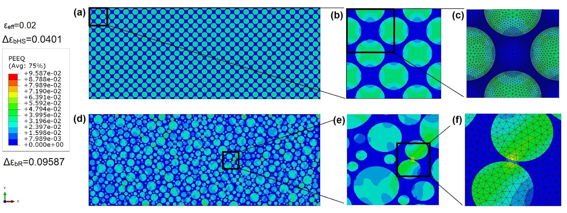


**Fig. S1.4** Distribution of accumulated effective plastic strains at a global strain $\boldsymbol{\varepsilon}_{\boldsymbol{eff}}=$0.02 in (a, b, c) bHS, and (d, e, f) bR materials; (b, c, e, f) show magnified areas outlined by respective black squares at lower-magnification distributions; FE meshes in deformed shapes are shown in (c, f) for the ease of CG / UFG phase identification. Colour codes on all the images correspond to a scale bar shown on the left-hand side along with reference directions and ranges between max-min plastic strain values in each bimodal material.

In the bR material, see **Figs. S1.4d**-**f**, accumulated effective plastic strains distribute extremely heterogeneously further increasing the range. In UFG regions, strains are predominantly elastic while sharp spikes to $\boldsymbol{\varepsilon}_{\boldsymbol{eff}}$ < 0.06 can be seen in the narrow ‘necks’ between CG areas. The latter are fully plastic with a strain gradients span ranging from $\boldsymbol{\varepsilon}_{\boldsymbol{eff}}=$0.095 in close proximity to the UFG ‘necks’ down to $\boldsymbol{\varepsilon}_{\boldsymbol{eff}}=$0.016 relatively far away from such.

***Stress-strain distributions at*** $\boldsymbol{\varepsilon}_{\boldsymbol{eff}}\boldsymbol{=}$***0.04 (strain concentrations in bR material)***

**Figs. S1.5a-c** reveal no principal changes in the distribution of von Mises stresses in the bHS material at this global strain level. At the same time, it shows that the local stress gradients at a unit cell scale evolve further to the level of $\Delta\sigma_{bHS}=$1294MPa, while their macroscopic distribution at the specimen-scale remains regular. The narrow high-stress pattern propagating through the UFG phase adjacent to CG areas in direction perpendicular to loading becomes continuous. The mean von Mises stress level in the CG phase increases to the level of 400MPa, while in the UFG phase it varies from 730MPa in the middle of skeleton nodes to 1600MPa in the ‘neck’ regions. These suggest that both the CG and UFG phases accumulated at least some level of plastic strain.

The bR material, see **Figs. S1.5d**-**f**, continue revealing no regular pattern in von Mises stress distribution with the range of stress variation now changing the earlier trend to decrease to $\Delta\sigma_{bR}=$1467 MPa. The high-stress regions in excess of 1700 MPa are now extended and can be found exclusively in the narrow UFG ‘necks’ between CG areas, while the level of stress in the latter occasionally exceeds 480 MPa. These can be interpreted as the indication of strain concentration, a presage of failure, in the UFG phases of the bR material.

**
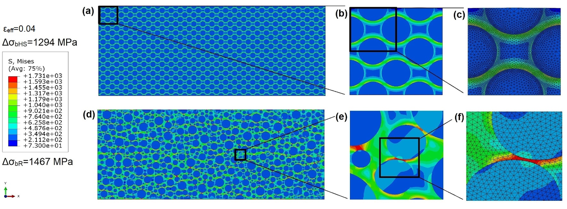
**

**Fig. S1.5** Distribution of von Mises stresses at a global strain $\boldsymbol{\varepsilon}_{\boldsymbol{eff}}=$0.04 in (a, b, c) bHS, and (d, e, f) bR materials; (b, c, e, f) show magnified areas outlined by respective black squares at lower-magnification distributions; FE meshes in deformed shapes are shown in (c, f) for the ease of CG / UFG phase identification. Colour codes on all the images correspond to a scale bar shown on the left-hand side along with reference directions and ranges between max-min stress values in each bimodal material.

**Figs S1.6a**-**c** suggest that in the bHS material the strain gradients at this global strain level continue evolving similar to the stress distribution. In the UFG skeleton, strains appear homogeneous exceeding slightly the elastic threshold of $\epsilon$*=*0.002. The CG areas appear to be fully plastic with accumulated strains varying from $\boldsymbol{\varepsilon}_{\boldsymbol{eff}}$ < 0.078 in the centre down to $\boldsymbol{\varepsilon}_{\boldsymbol{eff}}$ < 0.027 at their periphery. The ‘edge effect’ becomes distinct again generating higher strain levels in the narrow regions of top / bottom layers of CG areas propagating from higher-strained interiors on the specimen surfaces to the first sub-surface layer of UFG ‘necks’.


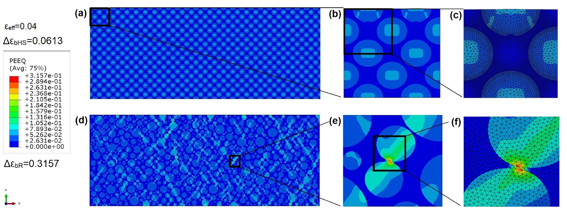


**Fig. S1.6** Distribution of accumulated effective plastic strains at a global strain $\boldsymbol{\varepsilon}_{\boldsymbol{eff}}=$0.04 in (a, b, c) bHS, and (d, e, f) bR materials; (b, c, e, f) show magnified areas outlined by respective black squares at lower-magnification distributions; FE meshes in deformed shapes are shown in (c, f) for the ease of CG / UFG phase identification. Colour codes on all the images correspond to a scale bar shown on the left-hand side along with reference directions and ranges between max-min plastic strain values in each bimodal material.

In the bR material, see **Figs. S1.6d**-**f**, accumulated effective plastic strains become having regular high-strain regions propagating through several CG / UFG areas at approximately 45° to loading direction. The range of accumulated plastic strain variation increases to $\Delta\boldsymbol{\varepsilon}_{bR}=$0.3157 with peak values in the narrowest UFG ‘necks’, see **Fig. S1.6f**. These severe plastic strain concentrations clearly indicate an approaching strain localisation also suggesting the beginning of bR specimen failure.

***Stress-strain distributions at*** $\boldsymbol{\varepsilon}_{\boldsymbol{eff}}\boldsymbol{=}$***0.1 (developed plastic deformation in bHS material)***

Stress-strain distributions at this and following global strain levels will only be considered for bHS sample still deforming homogeneously at macro-scale. **Figs. S1.7a-c** reveal that the local von Mises stress gradients at a unit cell scale evolve further $\Delta\sigma_{bHS}=$1481 MPa, while their macroscopic distribution at the specimen-scale remains regular. The continuous highest-stress pattern propagating through the UFG phase in loading direction now indicates narrow stress-drops around UFG ‘necks’ and top/bottom peaks of CG areas. The mean von Mises stress level in the CG phase increases to almost 500 MPa, while in the UFG phase it develops steeper gradient varying from 250 MPa to 1730 MPa. This suggests that both the CG and UFG phases are fully plastic now.


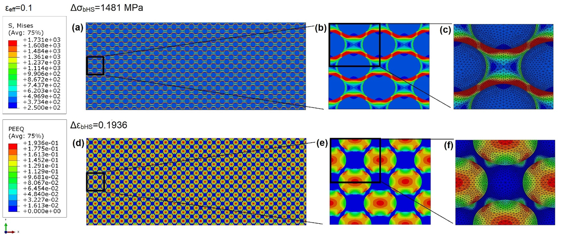


**Fig. S1.7** Distribution of von Mises stresses (a-c) and accumulated effective plastic strains (d-f) at a global strain $\boldsymbol{\varepsilon}_{\boldsymbol{eff}}=$ 0.1 in bHS material; (b, c, e, f) show magnified areas outlined by respective black squares at lower-magnification distributions; FE meshes in deformed shapes are shown in (c, f) for the ease of CG / UFG phase identification. Colour codes on all the images correspond to a scale bar shown on the left-hand side along with reference directions and ranges between max-min plastic strain values in each bimodal material.

The distribution of plastic strains in **Figs. S1.7d**-**f** indicate maximum values of $\boldsymbol{\varepsilon}_{\boldsymbol{eff}}$ ≈ 0.19 in the middle of CG areas and increasing local peaks on CG peripheries next to the UFG ‘necks’. These strain maxima are inter-connected by narrow lower-strain regions at $\boldsymbol{\varepsilon}_{\boldsymbol{eff}}$≈ 0.15 making cross-like pattern. In the rest of CG areas plastic strain levels decrease to $\boldsymbol{\varepsilon}_{\boldsymbol{eff}}$ ≈ 0.08. In the majority of UFG skeleton, plastic strains appear homogeneous and low still slightly exceeding the elastic threshold of $\epsilon$ = 0.002. At the same time, local spikes to the level of $\boldsymbol{\varepsilon}_{\boldsymbol{eff}}$ ≈ 0.08 can be found around the UFG ‘necks’ in the immediate vicinity of peripheral plastic strain peaks in the CG areas, see **Fig. S1.7f**. This pattern is still perfectly periodic and homogeneous macroscopically.

***Stress-strain distributions at*** $\boldsymbol{\varepsilon}_{\boldsymbol{eff}}\boldsymbol{=}$***0.174 (strain concentrations in bHS material)***

The macroscopic distribution of von Mises stresses at the specimen-scale appears to remain regular, see **Figs. S1.8a-c**, while local stress gradients at a unit cell scale reach $\Delta\sigma_{bHS}=$1563 MPa. The stress gradients are very sharp with two very small minima at the level of 170 MPa located symmetrically off-centre of UFG skeleton nodes. The continuous highest-stress pattern propagating through the UFG phase in loading direction appears thicker and more homogeneous still indicating some narrowing around top / bottom peaks of CG areas. In the CG phase, the mean von Mises stresses remain at approximately 500 MPa level, while highest values approaching 700 MPa are observed in the middle of CG areas and increasing local peaks on peripheries next to the UFG ‘necks’. Very narrow stress minima at the level of 170 MPa can also be found at left / right extremities of CG areas next to UFG skeleton regions narrowly stressed to 1300 MPa.


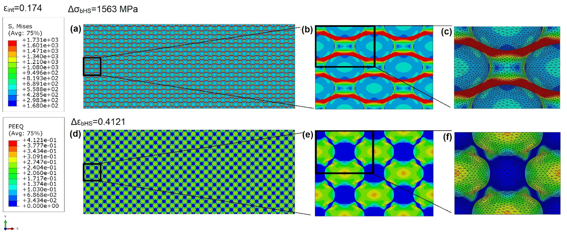


**Fig. S1.8** Distribution of von Mises stresses (a-c) and accumulated effective plastic strains (d-f) at a global strain $\boldsymbol{\varepsilon}_{\boldsymbol{eff}}=$ 0.174 in bHS material; (b, c, e, f) show magnified areas outlined by respective black squares at lower-magnification distributions; FE meshes in deformed shapes are shown in (c, f) for the ease of CG / UFG phase identification. Colour codes on all the images correspond to a scale bar shown on the left-hand side along with reference directions and ranges between max-min plastic strain values in each bimodal material.

The distribution of plastic strains in **Figs. S1.8d**-**f** reveals that the range of accumulated strains more than doubles with maximum values reaching $\boldsymbol{\varepsilon}_{\boldsymbol{eff}}$ ≈ 0.4121. At the same time, strain gradients become very sharp peaking on the CG peripheries next to the UFG ‘necks’ and slightly lower local maxima in the middle of the CG areas. In the rest of CG areas strain levels gradually decrease to $\boldsymbol{\varepsilon}_{\boldsymbol{eff}}\approx$0.07. In the majority of UFG skeleton accumulated strains do not change appreciably. However local spikes reaching $\boldsymbol{\varepsilon}_{\boldsymbol{eff}}\approx$0.27 and propagating on both sides of the UFG ‘necks’ almost close the gap between CG areas now, see **Fig. S1.8f**.

Such sharp gradients with very high amplitudes in both von Mises stress and accumulated plastic strain distributions indicate the beginning of bHS specimen failure.
