# Supplementary material for: Improvement of structural efficiency in metals by the control of topological arrangements in ultrafine and coarse grains
Source: Sci Rep. 2021 Aug 31;11:17445. doi: 10.1038/s41598-021-96930-3 (PMC8408239; doi:10.1038/s41598-021-96930-3)
Supplement: Supplementary file 2 — Supplementary Information 2. [file 41598_2021_96930_MOESM2_ESM.docx]

**Improvement of structural efficiency in metals
by the control of topological arrangements in ultrafine and coarse grains**

***Authors:*** Abdallah Shokry, Aylin Ahadi, Per Ståhle, Dmytro Orlov*
*Corresponding author, email: dmytro.orlov@material.lth.se.

Supplementary information S2

Comparison of three-dimensional, plane stress, plane strain and generalised plane strain cases in the finite-element simulations of bimodal harmonic-structure material

The choice to study a half-length specimen, split along the symmetry plane in the middle between the specimen ends, is the result of a compromise to keep the execution times reasonable, while still providing ample space for the undisturbed development of the localised straining after peak load. For example, a single square unit cell, as shown in **Fig. 10c**, requires 13’860 elements, while a geometrically equivalent single cube in a 3D-model with the same mesh settings demands over 1’300’000 elements. Even 1/8^th^ of the entire specimen model in 3D having 5×5×25 cubic unit cells would consist of more than 800 million elements, which for the tetrahedral element with 12 degrees of freedom would require an unacceptably large amount of memory. For instance, a mesh of just 15 million elements requires at least 170 GB in hard drive memory according evaluation by Abaqus/CAE. Therefore, a mesh of (only) 5.1 million elements was used for the 3D simulations for this Supplementary information, which is geometrically equivalent to 692’000 elements in 2D ^1^. In the 2D FE models finally used for the simulations in the main part of the manuscript, the total number of elements was 1’370’706 and 1’038’891 for the bHS and bR models, respectively.

For the harmonic-structure material, the mechanical state of the upper and the lower parts, split along $y=500$ μm, are identical before peak load. All meshed unit cells, at the same vertical position, as described in **Fig. 10** are exposed to identical mechanical load. This and the vertical symmetry plane across the centre of the unit cell makes the smallest identical periodic unit, a 50 μm wide and 500 μm high strip between the traction free surface and the mid-plane. Until peak load, the strip represents the mechanical state of all sections between the symmetry planes at $x=50$, 100, 150, etc. μm. The selected periodic cell covers $0\leq x\leq50$ and $0\leq y\leq500$ μm.

As can be vaguely observed in the subfigures of **Figs. S1.1** to **S1.6**, there are small differences in stresses and strains between the surface and parts below around 100 μm from the surface. Because of the vanishing tractions at the surface at $y=0$ and $y=1000$ μm, the surfaces are wavily contraction deformed with a 50 μm periodicity. The wavy deformation in the interior of the specimen decays with increasing distance from each surface. Still, it is anticipated that the stress perpendicular to the tensile direction is small.

Here, the 2D calculations are made on the selected $50\times500$ μm^2^ strip. It is assumed that plane stress, plane strain or generalised plane strain is established in the $z$-direction. The boundary conditions are constrained displacements $u_{x}=0$ at $x$ = 0, prescribed displacement $u_{x}=u_{o}$ at $x$ = 50 μm and constrained displacements in the $y$ direction at $y$ = 0. The surface at $y$ = 500 μm is traction free. All surfaces are free from shear tractions. The mesh consists of 13’860 six-node triangular isoparametric plane elements.

The 3D simulations in **Fig. S2.1** demonstrate the lowest maximum tensile strength along with the lowest uniform elongation. The 2D plane strain and generalised plane strain obviously impose constraints that are believed to exaggerate the structural strength. Because of the limited width, the behaviour after the peak load is very different from that of a wider mesh where there is ample space to an unconstrained evolution of localised strain. When these simulations are compared to nearest data from actual experimental stress-strain curves ^2^, the later demonstrate peak stress in the range between the simulated 3D and plane-stress simulations along with slightly larger uniform elongation.


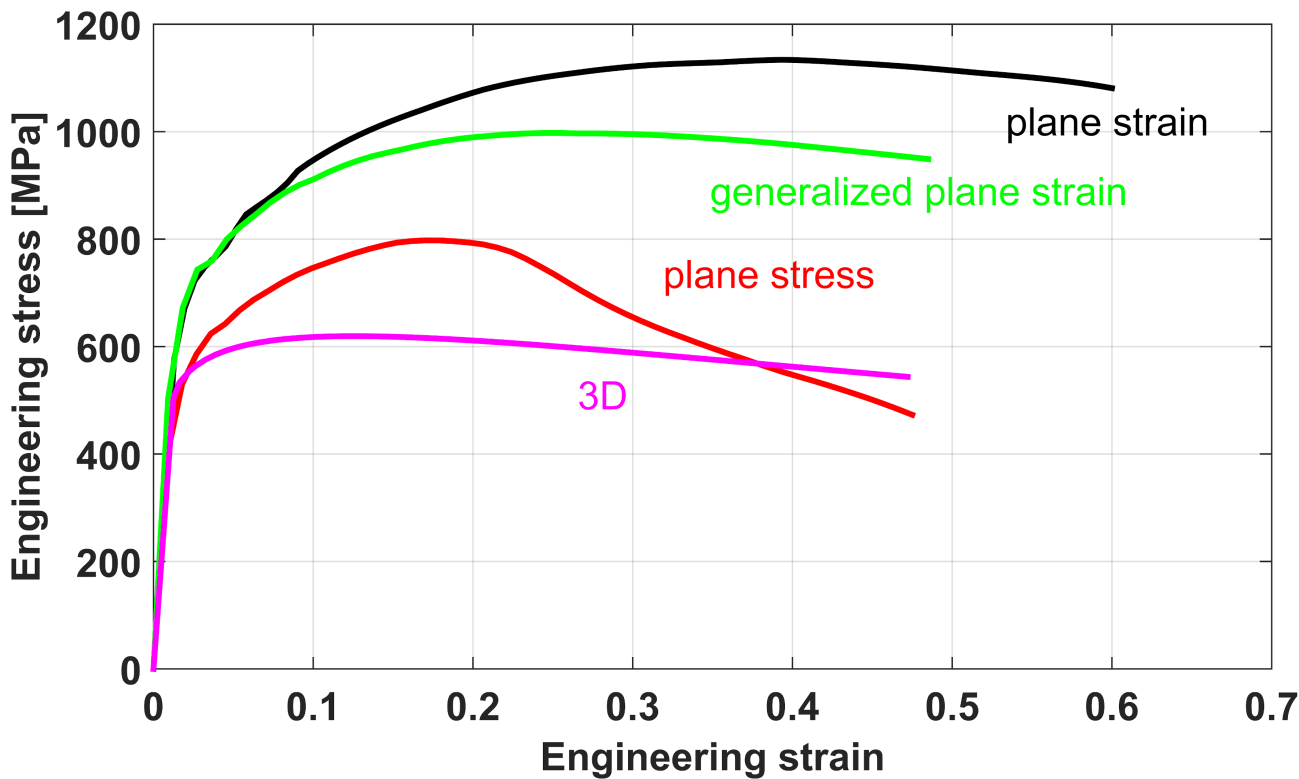


**Fig. S2.1**. Engineering effective tensile stress for three-dimensional (3D) FE model and assumed plane stress, plane strain and generalised plane strain conditions in the $z$-direction. Calculations in 2D are made on 500 μm $y$-direction strips with 50 μm $x$-direction width. Tension is applied in the $x$-direction. The behaviour after peak load is not representative of a full-size specimen.

The plane stress model is in general known to be less stiff than plane strain models because of the loss of the constrained cross section deformation. The z-direction normal strain that has to be independent of the x and y coordinates for the plane strain models does not apply to the plane stress. This plane stress has in common with the 3D model which for both leads to a significant reduction of its in-plane stiffness. Thus, a plausible reason for the deviating 2D plane strain models is the extrusion mimicking and the cross plane deformation constraint.

The 2D axisymmetric model might also be of interest. However, preparing specimens of respective geometry for real-life experiments is more complex. Corresponding experimental data for benchmarking were not available. All relevant experimental works of interest are reported on ‘flat’ dog-bone shaped specimens. Therefore, 2D axisymmetric model was not considered in the present paper but should be the subject of a separate study in the future.

Based on these results, it is believed that the most conservative 2D ‘plane stress’ model is most appropriate for the modelling at present. Therefore, it was decided to select it for the simulations and to limit the latter to primarily qualitative analysis due to complexity and cross-scale character of the problem. The fine-tuning of the model and quantitative analysis are reserved for future studies. They will be reported in a follow-up publication.

Accuracy of calculations

A series of calculations was carried out on a unit cell to establish convergence. The calculations were made with meshes ranging from 1544 to 5448 finite elements covering a 0.1x0.1mm^2^ periodic cell of the bHS material. For the full model this corresponds to the range 386 000 to 1 362 000 elements. The unit cell is stretched uniaxially to 0.4%. The calculation then covers the elastic-plastic behaviour prior to global strain localisation. Table S2.1 shows the largest effective von Mises stress obtained for different mesh densities, cf. Table S2.1.

**Table S2.1**. Largest von Mises effective stress at 0.4% uniaxial stretching. Percentages are deviations from the average peak stress 1538 MPa.

| No. of elements | Stress [MPa] | Deviation |
| --- | --- | --- |
| 1544 | 1545 | 0.5% |
| 2460 | 1528 | -0.6% |
| 3066 | 1553 | 1.0% |
| 4012 | 1536 | -0.1% |
| 5448 | 1528 | -0.6% |

The results do not show any particular trend but rather fluctuates between -0.6% to +1% around an average value of 1538MPa. The lack of any visible correlation with the number of elements and the seemingly random scatter lead us to assume that all meshes fulfil the requirements for convergence. To make the most of it, a dense mesh with around 5500 elements per periodic cell that gives 1.37 million elements was selected for the full model calculations.

Further, the scatter between -0.6% to 1% is an expected variation that is caused by the free mesh generated by a Delaunay type of triangulation^3^. The algorithm used by a preprocessor for the FEM code Abaqus/CAE leads to uncorrelated local changes of the mesh that comes with rather small changes of the required densities and gradients.

**References**

1 Ibishi, B. *Finite-Element Simulations of Harmonic Structured Materials* MSc thesis, Lund University, (2016).

2 Orlov, D., Zhou, J., Hall, S., Ota-Kawabata, M. & Ameyama, K. Advantages of architectured harmonic structure in structural performance. *IOP Conference Series: Materials Science and Engineering* **580**, 265-272, doi:10.1088/1757-899x/580/1/012019 (2019).

3 Delaunay, B. Sur la sphère vide. A la mémoire de Georges Voronoï. *Bulletin de l'Académie des Sciences de l'URSS. Classe des sciences mathématiques et naturelles*, 793–800 (1934).
